# Oxygen dependence of metabolic fluxes and energy generation of *Saccharomyces cerevisiae *CEN.PK113-1A

**DOI:** 10.1186/1752-0509-2-60

**Published:** 2008-07-09

**Authors:** Paula Jouhten, Eija Rintala, Anne Huuskonen, Anu Tamminen, Mervi Toivari, Marilyn Wiebe, Laura Ruohonen, Merja Penttilä, Hannu Maaheimo

**Affiliations:** 1 VTT Technical Research Centre of Finland, Espoo, Finland

## Abstract

**Background:**

The yeast *Saccharomyces cerevisiae *is able to adjust to external oxygen availability by utilizing both respirative and fermentative metabolic modes. Adjusting the metabolic mode involves alteration of the intracellular metabolic fluxes that are determined by the cell's multilevel regulatory network. Oxygen is a major determinant of the physiology of *S. cerevisiae *but understanding of the oxygen dependence of intracellular flux distributions is still scarce.

**Results:**

Metabolic flux distributions of *S. cerevisiae *CEN.PK113-1A growing in glucose-limited chemostat cultures at a dilution rate of 0.1 h^-1 ^with 20.9%, 2.8%, 1.0%, 0.5% or 0.0% O_2 _in the inlet gas were quantified by ^13^C-MFA. Metabolic flux ratios from fractional [U-^13^C]glucose labelling experiments were used to solve the underdetermined MFA system of central carbon metabolism of *S. cerevisiae*.

While ethanol production was observed already in 2.8% oxygen, only minor differences in the flux distribution were observed, compared to fully aerobic conditions. However, in 1.0% and 0.5% oxygen the respiratory rate was severely restricted, resulting in progressively reduced fluxes through the TCA cycle and the direction of major fluxes to the fermentative pathway. A redistribution of fluxes was observed in all branching points of central carbon metabolism. Yet only when oxygen provision was reduced to 0.5%, was the biomass yield exceeded by the yields of ethanol and CO_2_. Respirative ATP generation provided 59% of the ATP demand in fully aerobic conditions and still a substantial 25% in 0.5% oxygenation. An extensive redistribution of fluxes was observed in anaerobic conditions compared to all the aerobic conditions. Positive correlation between the transcriptional levels of metabolic enzymes and the corresponding fluxes in the different oxygenation conditions was found only in the respirative pathway.

**Conclusion:**

^13^C-constrained MFA enabled quantitative determination of intracellular fluxes in conditions of different redox challenges without including redox cofactors in metabolite mass balances. A redistribution of fluxes was observed not only for respirative, respiro-fermentative and fermentative metabolisms, but also for cells grown with 2.8%, 1.0% and 0.5% oxygen. Although the cellular metabolism was respiro-fermentative in each of these low oxygen conditions, the actual amount of oxygen available resulted in different contributions through respirative and fermentative pathways.

## Background

The yeast *Saccharomyces cerevisiae *is a facultative anaerobic organism. It is able to respond to external oxygen availability by utilizing both respirative and fermentative metabolic modes and it grows at a fast rate even when aerobic respiration is limited or completely prevented [1-3]. Metabolic response to oxygen availability requires alteration of the intracellular fluxes. The intracellular flux distribution alterations in general are mediated through transcriptional, protein level and metabolic regulation, the fluxes being the integrated network response of the regulated interactions between enzymes and metabolites [4]. Oxygen is a major determinant of the physiology of *S. cerevisiae *but understanding of the oxygen dependence of intracellular metabolic flux distributions is still scarce. Furthermore the dependence of the flux distribution on oxygen availability is of great interest in many biotechnological applications of *S. cerevisiae*, particularly those requiring a low oxygen concentration to obtain maximal product yield with simultaneous limited side products, including biomass [5].

During aerobic growth oxygen serves as a final electron acceptor in respiration. When oxygen availability is limited, cells need alternative acceptors for the electrons of NADH and FADH_2 _to maintain the redox balance. In aerobic conditions the assimilatory NADH is oxidised mainly by the external NADH dehydrogenases or transported into mitochondria by the glycerol-3-phosphate shuttle, whereas in the absence of oxygen *S. cerevisiae *produces glycerol as a redox sink [6,7]. Since glycerol production leads to net hydrolysis of ATP and loss of carbon, *S. cerevisiae *uses oxygen preferentially for oxidation of assimilatory NADH when oxygen availability is restricted [8,9]. In addition the oxidative stress to which the cells are exposed in high external oxygen availability imposes other redox challenges.

When external oxygen availability is limited *S. cerevisiae *generates energy partially or completely through fermentation, although it is less energy efficient than respiratory metabolism [2]. While the high fermentative capacity enables *S. cerevisiae *to produce energy at a sufficient rate even in anaerobic conditions [10], constant anaerobic growth requires addition of unsaturated fatty acids and ergosterol to the culture medium since oxygen is an essential reactant in sterol biosynthesis and anabolic desaturation reactions [1,11]. Furthermore, when the respiratory system coupling NADH oxidation to the generation of a proton gradient across the mitochondrial membrane is limited, additional means for cross-membrane transport of metabolites and ions are required [1]. Growth when there is limited or no aerobic respiration thus requires an adjusment of metabolism and a major redistribution of metabolic fluxes compared to fully respiratory metabolism.

Respiration of *S. cerevisiae *becomes restricted, not only when oxygen availability is limited, but also in fully aerobic conditions when there is an excess of repressive carbon source [12-16]. The excess repressive carbon source mediates complex transcriptional regulation, including repression of respiratory genes, and thus lowers the maximal respiratory rate. Limited respiratory capacity results in alcoholic fermentation [17]. Aerobic alcoholic fermentation is also triggered at high growth rates in aerobic chemostats [18,2]. The limited respiratory capacity in both conditions has been shown to result in redistribution of intracellular carbon fluxes through respiratory and fermentative pathways [19,20,18].

Intracellular metabolic flux distributions are determined by metabolic flux analysis (MFA) which is based on stoichiometric modeling, with a system of mass balance equations for intracellular metabolites [21]. Usually the mass balance equation system is underdetermined since the number of degrees of freedom exceeds the number of measured extracellular fluxes. Linear programming can be used to solve the MFA system if a biologically meaningful objective function is formulated [22]. Including redox cofactors that are involved in all cellular metabolism into the mass balancing renders the system more constrained but requires detailed knowledge on the cofactor specificities of different isoenzymes and the relative activities of the isoenzymes in the conditions studied. This information is rarely available in the extent of a genome wide metabolic network. However, MFA with additional experimental constraints from ^13^C-labelling experiments combined with mass spectrometry (MS) or nuclear magnetic resonance spectroscopy (NMR) detection of labelling patterns in metabolic compounds [4,18,19,23] can be used to resolve intracellular fluxes through complex pathway structures [24], including compartmentalised eukaryotic metabolic networks [25-27]. The established knowledge on the topology of the metabolic network of *S. cerevisiae *[28,29] enables modelling for MFA. The distribution of intracellular fluxes of *S. cerevisiae *to respirative and fermentative pathways in response to different reduced oxygen provisions has not been quantified with MFA combined with ^13^C-tracer experiments before. ^13^C-labelling has previously been used to quantify the redistribution of fluxes in *S. cerevisiae *to the respirative and fermentative pathways in response to glucose repression by comparison of batch culture fluxes to glucose-limited derepressed chemostat culture fluxes at low growth rate [19,20] and in response to high growth rates in aerobic chemostat cultures [18].

The physiology of *S. cerevisiae *in aerobic and in anaerobic conditions has been studied at the levels of gene expression [30,31], metabolite concentrations and enzyme activities [32,33], by the means of ^13^C-metabolic flux ratio (METAFoR) analysis [20,34], metabolic flux analysis (MFA) [9,35,36] and regulation analysis [37]. ^13^C-tracer experiments in combination with metabolic flux analysis (MFA) have previously been applied only in studying the flux distributions of *S. cerevisiae *in aerobic glucose-limited chemostat cultures [19,38]. The effect of intermediate oxygenation conditions on *S. cerevisiae *metabolism has been the subject of some classical studies [2,3,39], including studies of the dependence of gene expression in *S. cerevisiae *on oxygenation through heme-dependent and heme-independent regulation networks, reviewed by Zitomer and Lowry (1992) and Kwast *et al. *(1998) [40,41]. Oxygen dependent transcriptional responses were observed in a range of oxygen concentrations. In addition Franzén (2003) studied ethanol production and metabolic fluxes of *S. cerevisiae *in respiratory quotient (RQ) controlled continuous cultures in a number of different microaerobic conditions by MFA without ^13^C-tracers [9]. Franzén observed a positive correlation between biomass generation and reoxidisation of assimilatory NADH, indicating the importance of the redox balance as a determinant of the metabolic flux distribution.

The work presented here is the first where the intracellular metabolic flux distributions of *S. cerevisiae *in different levels of low external oxygen in chemostat cultures at low growth rate were quantified using ^13^C-labelling. The low growth rate, 0.1 h^-1^, ensured that the metabolic effects observed stemmed solely from the reduced availability of oxygen, rather than from exceeding the respiratory capacity. The flux distributions of *S. cerevisiae *central carbon metabolism under five different oxygenation conditions were solved by combining the metabolic pathway branching point constraints from the ^13^C-labelling experiments with metabolite balancing using MFA. By including the additional constraints from the ^13^C-labelling experiments, the cofactors could be left out from the metabolite balancing in MFA and thus the redox status regulated carbon fluxes could be reliably assessed. Completely respirative metabolism was observed in fully aerobic conditions and fully fermentative metabolism in anaerobic conditions and in the three different reduced oxygenation conditions the actual amount of oxygen available was observed to result in different flux contributions through respirative and fermentative pathways. Based on the flux distributions, energy generation of *S. cerevisiae *in the different oxygenation conditions was also determined. This paper also compares the metabolic flux distribution in different conditions of oxygen provision with the transcriptional levels of a number of metabolic genes in the same conditions, as published recently [32].

## Results

**Figure 1:**
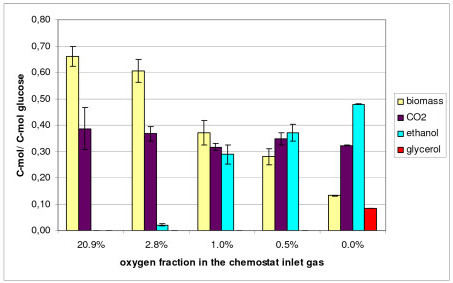
**Average yields in *S. cerevisiae *CEN.PK113-1A glucose-limited chemostat (D = 0.1 h^-1^) cultures**. Average yields of biomass (41.6 C-mmol g biomass^-1 ^[19]), CO_2_, ethanol and glycerol on glucose (C-mol/C-mol) in the [U-^13^C]glucose labelled replicate cultivations of *S. cerevisiae *CEN.PK113-1A in glucose-limited chemostat (D = 0.1 h^-1^) in different oxygenation conditions: 20.9%, 2.8%, 1.0%, 0.5% and 0.0% oxygen of the chemostat inlet gas.

*S. cerevisiae *CEN.PK113-1A was grown in glucose-limited chemostats at a dilution rate of 0.1 h^-1 ^in five different oxygenation conditions (20.9%, 2.8%, 1.0%, 0.5% and 0.0% O_2 _in the inlet gas). The corresponding average specific oxygen uptake rates (OUR) at these oxygen concentrations were 2.7, 2.5, 1.7, 1.2, and 0.0 mmol O_2 _g biomass^-1 ^h^-1 ^as derived from a number of replicate chemostat cultivations [32]. The specific uptake rate of glucose, excretion rates of acetate, ethanol and glycerol and the biomass concentration in the different oxygenation conditions in the ^13^C labelled replicate cultivations are given in Table 1. Net ethanol production was not observed in the aerobic cultures provided with 20.9% oxygen, indicating a fully respiratory metabolism. In 2.8% oxygen, slight ethanol excretion was observed indicating a shift to respiro-fermentative metabolism. In lower oxygen conditions, ethanol excretion rates increased further and the highest ethanol excretion rate was observed in anaerobic conditions, in which the metabolism was completely fermentative. As expected [42], the concentration of biomass was five times lower in anaerobic than in fully aerobic cultivations. Net production of glycerol was observed only in anaerobic cultivations. When only 0.5% oxygen was provided, ethanol and CO_2 _yields exceeded the yield of biomass (Figure 1). In anaerobic conditions the biomass yield was only one fourth of the yield of the main product ethanol. The carbon balances closed between 96–113% in all the cultures (41.6 C-mmol/g DW [19]).

**Table 1:** **Uptake and production rates and biomass concentration in *S. cerevisiae *CEN.PK113-1A chemostat cultures**. Glucose uptake rate, glycerol, acetate and ethanol production rate and biomass concentration (mean ± SEM) of the ^13^C-labelled glucose-limited chemostat cultures (D = 0.10 h^-1^, pH 5.0, 30°C, 1,5 vvm gas flow) of *S. cerevisiae *CEN.PK113-1A used as input values in the ^13^C-MFA.

	O_2 _provided									
	
	20.9%		2.8%		1.0%		0.5%		0.0%	
					
	I	II	I	II	I	II	I	II	I	II
Glucose uptake rate [mmol/(g DW)^-1^h^-1^]	1.28 ± 0.04	0.87 ± 0.04	1.36 ± 0.04	1.28 ± 0.02	1.97 ± 0.02	2.13 ± 0.06	2.20 ± 0.09	2.78 ± 0.16	6.30 ± 0.25	6.58 ± 0.16
Glycerol production rate [mmol/(g DW)^-1^h^-1^]	0.00 ± 0.00	0.00 ± 0.00	0.00 ± 0.00	0.00 ± 0.00	0.00 ± 0.00	0.00 ± 0.00	0.00 ± 0.00	0.00 ± 0.00	1.05 ± 0.01	1.11 ± 0.03
Acetate production rate [mmol/(g DW)^-1^h^-1^]	0.00 ± 0.00	0.00 ± 0.00	0.00 ± 0.00	0.00 ± 0.00	0.00 ± 0.00	0.00 ± 0.00	0.00 ± 0.00	0.00 ± 0.00	0.00 ± 0.00	0.00 ± 0.00
Ethanol production rate [mmol/(g DW)^-1^h^-1^]	0.00 ± 0.00	0.00 ± 0.00	0.07 ± 0.00	0.10 ± 0.01	1.56 ± 0.02	2.00 ± 0.08	2.59 ± 0.09	2.91 ± 0.18	9.05 ± 0.21	9.47 ± 0.31
Biomass (g DW l^-1^)	5.17 ± 0.04	5.31 ± 0.12	4.61 ± 0.09	4.86 ± 0.05	3.22 ± 0.02	2.70 ± 0.03	2.21 ± 0.03	2.08 ± 0.03	1.03 ± 0.02	0.98 ± 0.02

**Figure 2:**
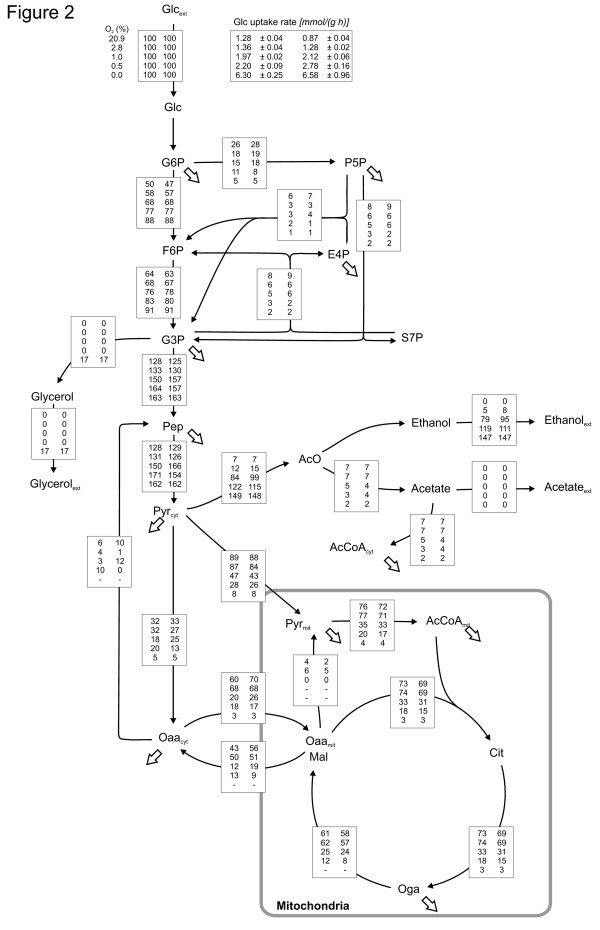
**Metabolic net flux distributions of *S. cerevisiae *CEN.PK113-1A in different oxygenation conditions**. Net flux distribution of *S. cerevisiae *CEN.PK113-1A in glucose-limited chemostat, D = 0.1 h^-1^, in different oxygenation conditions: 20.9%, 2.8%, 1.0%, 0.5% and 0.0% oxygen of the chemostat inlet gas. The net fluxes are shown as relative fluxes normalised to the specific glucose uptake rate in the corresponding experiment. The fluxes for each reaction in the model corresponding to the 20.9%, 2.8%, 1.0%, 0.5% and 0.0% oxygen of the chemostat inlet gas are given from top to bottom and the flux values from replicate experiment are given from left to right. The specific glucose uptake rates corresponding to the different oxygenation conditions and the replicate experiments are given at the top of the figure. The net flux distributions were determined using fractional [U-^13^C]glucose feed and metabolic flux ratio (METAFoR) analysis to obtain additional experimental constraints to render an underdetermined metabolite mass balance system solvable. The Matlab function *fmincon *performing nonlinear optimisation was applied to solve the net fluxes.

Metabolic flux ratios were determined by METAFoR analysis from the fractionally ^13^C-labelled biomass hydrolysates by 2D NMR [34,43]. The flux ratios were calculated from the relative abundances of intact carbon backbone fragments, fragmentomers, in proteinogenic amino acids originating from a single carbon source molecule of glucose, determined from the ^13^C-fine structures in 2D NMR spectra (Additional file 1). Flux ratios of metabolic branching points in the central carbon metabolism of *S. cerevisiae *in the different oxygenation conditions are given in Table 2. In ^13^C-MFA metabolic flux ratios from the METAFoR analysis were used as additional constraints in a MFA system to be able to solve the metabolic net flux distribution without including the cofactors NADH and NADPH or ATP in the metabolite mass balances. The metabolic net fluxes in the different oxygenation conditions are shown in Figure 2. The confidence intervals (95%) for the net fluxes from Monte Carlo simulations of noise to the flux ratio and extracellular flux rate input data are included in Additional file 2.

**Table 2:** **Metabolic flux ratio (METAFoR) analysis results**. Metabolic flux ratio (METAFoR) analysis results showing the origins of metabolic intermediates during growth of *S. cerevisiae *CEN.PK113-1A in glucose-limited, ^13^C-labelled chemostat cultures (D = 0.1 h^-1^) at different fractions of oxygen in the chemostat inlet gas. Values for two independent cultivations are given for each condition.

Metabolite	% fraction of total pool									
	
	20.9% O_2_		2.8% O_2_		1.0% O_2_		0.5% O_2_		0.0% O_2_	
					
	I	II	I	II	I	II	I	II	I	II
Pep from pentose phosphates (ub)^a^	30 ± 9	34 ± 11	19 ± 6	20 ± 7	15 ± 6	19 ± 7	10 ± 7	6 ± 9	4 ± 4	4 ± 5
P5P from G3P and S7P (transketolase reaction)	51 ± 3	56 ± 6	64 ± 5	63 ± 4	82 ± 3	77 ± 3	74 ± 6	79 ± 4	86 ± 3	86 ± 5
P5P from E4P (transketolase and transaldolase)	34 ± 2	35 ± 2	27 ± 2	25 ± 2	28 ± 2	24 ± 2	26 ± 2	38 ± 2	14 ± 2	15 ± 2
Ser from Gly and C1-unit	62 ± 4	61 ± 4	61 ± 4	61 ± 4	63 ± 3	62 ± 3	62 ± 4	58 ± 3	57 ± 3	58 ± 3
Gly from CO_2 _and C1-unit	4 ± 4	3 ± 3	5 ± 3	6 ± 3	4 ± 3	4 ± 3	0 ± 4	4 ± 3	4 ± 3	2 ± 3
Pep from Oaa_cyt _(PEPck)	4 ± 7	7 ± 8	3 ± 6	1 ± 6	2 ± 10	7 ± 10	6 ± 12	0 ± 14	nd	nd
Oaa_mit _from Pep	30 ± 2	31 ± 2	30 ± 2	29 ± 2	34 ± 2	38 ± 2	48 ± 2	57 ± 2	100 ± 2	100 ± 2
Oaa_mit _from Oaa_cyt_	50 ± 3	55 ± 4	52 ± 4	54 ± 3	45 ± 2	51 ± 2	60 ± 3	69 ± 2	99 ± 2	99 ± 2
Oaa_cyt _from Pep	43 ± 2	37 ± 3	39 ± 3	35 ± 2	61 ± 3	57 ± 3	62 ± 4	59 ± 4	nd	nd
Oaa_cyt _reversibly converted to fumarate	10 ± 7	18 ± 17	19 ± 7	17 ± 10	6 ± 5	10 ± 9	8 ± 5	14 ± 4	18 ± 7	21 ± 3
Oaa_mit _reversibly converted to fumarate	64 ± 15	77 ± 17	71 ± 15	61 ± 14	60 ± 13	60 ± 13	62 ± 11	70 ± 8	29 ± 4	27 ± 4
Pyr_mit _from malate (ub)^a^	3 ± 3	2 ± 6	4 ± 4	4 ± 3	0 ± 4	0 ± 4	nd^b^	nd	nd	nd
Pyr_mit _from malate (lb)^a^	2 ± 2	1 ± 4	3 ± 3	3 ± 2	0 ± 2	0 ± 2	nd	nd	nd	nd

^a^ ub upper bound, lb lower bound for the fraction of total pool, see Methods for details

^b^ nd not determined, see Methods for details

### Glycolytic and PPP fluxes

**Figure 3:**
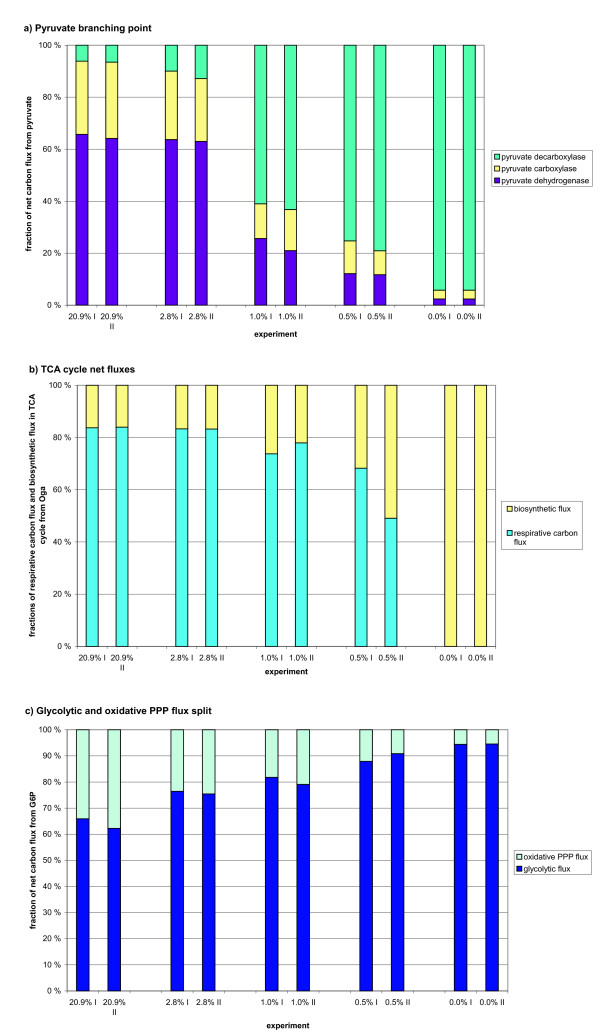
**Fractional distributions of carbon fluxes in metabolic branching points**. Fractional distribution of carbon fluxes a) from the pyruvate branching point, b) in the TCA cycle and c) from G6P to glycolysis and PPP in *S. cerevisiae *CEN.PK113-1A in glucose-limited chemostats, at D = 0.1 h^-1^, in 20.9%, 2.8%, 1.0%, 0.5% and 0.0% oxygen of the chemostat inlet gas. Replicate experiments are indicated with I and II.

The METAFoR analysis showed that in fully aerobic conditions on average 32% or less of Pep originated from the PPP and the combined pool of pentose phosphates (Table 2). In lower oxygen conditions, the relative PPP flux was lower and even with 2.8% oxygen the fraction of Pep originating from pentose phosphates was only 20%. The carbon flux split ratio from G6P to glycolysis and to the oxidative branch of PPP is shown in Figure 3. The relative flux from G6P to the PPP pathway decreased as the oxygen provision was reduced. However, the results of ^13^C MFA, shown in Figure 2, revealed that this decrease of the relative PPP flux was a result of increased glycolytic flux, while the specific flux through the oxidative branch of the PPP remained relatively constant. Progressively higher glycolytic fluxes were observed in 1.0% and in 0.5% oxygen. In anaerobic conditions the net flux in lower glycolysis remained almost the same as in 0.5% oxygen since a fraction of the carbon flux was lost in upper glycolysis to glycerol production. In anaerobic conditions the PPP flux contribution to Pep could be somewhat overestimated since the contribution of the phosphoenolpyruvate carboxykinase (PEPck) reaction to the labelling status of Pep was assumed insignificant. The flux ratio of PEPck contribution to Pep was generally lower when oxygen provision was lower.

The METAFoR analysis also gives insight into the reversible reactions of transketolase, TK, and transaldolase, TA, (Table 2), since these reactions cleave the carbon backbone of the pentose phosphates in specific locations [43]. Higher fractions of pentose phosphates showing the reversible action of a transketolase reaction were observed when less oxygen was provided than with more. There was no clear trend in the flux through the reversible transaldolase reaction, but it was low in anaerobic cultures compared to the other conditions. The high fraction of pentose phosphates cleaved by TK and TA may reflect the proposed ping-pong mechanism of these enzymes, allowing the reaction to proceed backwards before releasing the keto- or aldogroup [44].

### Anaplerosis and the Pyr branching point

The relative anaplerotic flux, the anaplerotic flux ratio defined here as the fraction of Oaa_mit _molecules originating from Pep, was on average 31% and 30% in 20.9% and 2.8% oxygen respectively, while the relative flux from pentose phosphates to Pep was lower in 2.8% oxygen than in fully aerobic conditions (Table 2). In 1.0% oxygen the anaplerotic flux ratio was slightly higher (36%), and it was clearly higher in 0.5% oxygen (53%) than in fully aerobic conditions. Thus, in 0.5% oxygen approximately half of the Oaa_mit _molecules originated from anaplerosis.

The carbon flux from Pyr branches into three pathways through pyruvate dehydrogenase, pyruvate decarboxylase and pyruvate carboxylase. The pyruvate dehydrogenase reaction is the first step for the carbon flux directed to the TCA cycle. The pyruvate decarboxylase reaction is the starting point for a fermentative pathway, and pyruvate carboxylase catalyses the carboxylation of Pyr to Oaa and thus also carries the anaplerotic flux. The carbon flux distributions at the pyruvate branching point were similar in fully aerobic conditions and in 2.8% oxygen (Figures 2 and 3). Slightly lower fluxes through pyruvate dehydrogenase and pyruvate carboxylase were observed in 2.8% oxygen than in 20.9% oxygen corresponding to the higher carbon flux through pyruvate decarboxylase, which reflected the production of ethanol. In conditions receiving less than 2.8% oxygen the carbon fluxes were redistributed at the pyruvate branching point and fermentative fluxes became dominating. In 1.0% oxygen the major carbon flux (on average 62%) from the pyruvate branching point was directed through pyruvate decarboxylase. The corresponding value in 0.5% oxygen was on average 77%. In 1.0% oxygen the fraction of carbon flux through pyruvate dehydrogenase (on average 23%) was less than half that observed in fully aerobic conditions (on average 65%), while the fraction of carbon flux through pyruvate carboxylase was 15% compared to 29% in fully aerobic conditions. In anaerobic conditions 94% of the carbon flux from the pyruvate branching point was directed through pyruvate decarboxylase, while pyruvate dehydrogenase flux contributed only 2% of the total flux (Figure 3).

### TCA cycle fluxes

**Figure 4:**
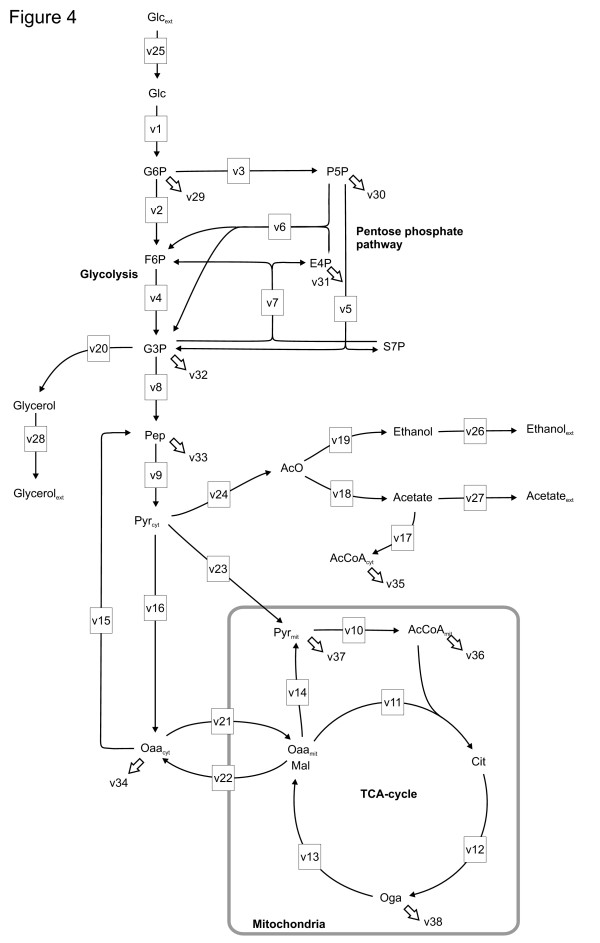
**Metabolic network model of the central carbon metabolism of *S. cerevisiae***. Metabolic network model of the central carbon metabolism of *S. cerevisiae *was applied in the ^13^C-metabolic flux analysis for determination of net fluxes in different oxygenation conditions. The cytosolic and mitochondrial compartments and glycolytic, pentose phosphate, TCA cycle and fermentative pathways were included in the model. The fluxes are presented as net fluxes and the directions of the arrows represent the directions of positive net fluxes. The compounds consumed or produced by external fluxes are denoted with a subscript ext. The anabolic reactions from metabolic intermediates to biosynthesis are represented by small arrows.

Low net TCA cycle fluxes were observed in low oxygen concentrations. 2.8% oxygen in the chemostat inlet gas was enough to maintain the net TCA cycle flux at a level almost as high as in the fully aerobic conditions, but progressively lower fluxes were observed when less oxygen was provided (Figure 2). Limitation in oxygen availability reduced the respirative carbon flux through the TCA cycle, the net flux from Oga through the TCA cycle to Oaa_mit _(*x*_13_, Figure 4), whereas the specific biosynthetic flux from the TCA cycle remained constant (*x*_38_, Figure 4). In fully aerobic conditions the respirative carbon flux from Oga was 84% of the net flux and even in 0.5% oxygen the respirative carbon flux was the major fraction of the net carbon flux in the TCA cycle (on average 59%) (Figure 3).

In anaerobic conditions, the C2–C3 fragments in Pep, Oaa_cyt _and Oaa_mit _had equal labelling patterns, as deduced from the corresponding amino acid labelling patterns (see the fragmentomer data in Additional file 1). Thus, anaplerotic flux was the only source of Oaa_mit _(Table 2) and the TCA cycle operated as a branched pathway, with oxidative and reductive branches, instead of as a cycle [45]. The equal fractions of intact C2–C3 fragments in Pep, Oaa_cyt _and Oaa_mit _result in unresolved flux ratios at the metabolic branching points of Pep, Oaa_cyt _and Pyr_mit_. Therefore, the metabolic network model was simplified by neglecting the PEPck activity and resolving only the net transfer of Oaa across the mitochondrial membrane at the Oaa branching point. The contribution of malic enzyme flux to the Pyr_mit _pool could not be quantified in anaerobic conditions, because the carbon fragments from the malic enzyme flux would have the same labelling pattern as the carbon fragments originating from Pyr_cyt_. However, when 0.5% oxygen was provided, no contribution for malic enzyme flux could be observed. In anaerobic conditions, symmetrisation of Oaa as the result of reversible exchange with fumarate was observed, but this label-scrambling flux could not be quantified with the current experimental set up. The labelling pattern of Oaa_mit _could only be partly determined from the amino acids, in which the carbon backbone originates from Oga (Table 2).

### Energy metabolism

In anaerobic conditions, where no aerobic respiration is taking place, ATP is generated solely through substrate level phosphorylations. Fermentation allows redox neutral anaerobic ATP generation when acetaldehyde acts as an electron acceptor for NADH. The biosynthetic ATP requirement per biomass unit was estimated from the anaerobic ATP generating and consuming net fluxes. The specific biosynthetic ATP requirements were assumed to be constant in all oxygenation conditions, since biomass composition was assumed to be constant, as indicated in previous experimental observations which showed essentially the same biomass composition in the two extreme conditions, i.e. in fully aerobic and in anaerobic conditions [19,35]. ATP generation through substrate level phosphorylations was calculated from the reaction stoichiometry and the net flux data. The rest of the ATP demand was assumed to be provided by respirative ATP generation. The fraction of ATP generated through respiration to meet the ATP demand was 59% in fully aerobic conditions and decreased with decreasing oxygenation, as ethanol production increased (Table 3). In 0.5% oxygen, 25% of the ATP was still generated through respiration.

**Table 3:** Energetic features in different oxygenation conditions. Energetic features of *S. cerevisiae *CEN.PK113-1A grown in glucose-limited chemostat, D = 0.1 h-1 in different oxygenation conditions.

	O_2 _provided in fermentor inlet gas				
	
	20.9%	2.8%	1.0%	0.5%	0.0%
ATP from respiration (%)	59	55	36	25	0
OUR (mmol g^-1^h^-1^)	2.7	2.5	1.7	1.2	0
ATP/O^a^	0.9	1.0	1.1	1.1	-
ATP/2e^-b^	1.0	0.9	1.0	1.2	-

^a^Calculated from the oxygen uptake rate (OUR).

^b^Calculated from the flux of electron donors to the respiratory chain.

The efficiency of oxidative phosphorylation in different levels of oxygen was assessed by determining the P/O ratios in the different conditions. By neglecting the fraction of oxygen consumed in pathways other than respiration [46], P/O ratios were estimated from the measured OURs and the estimated amount of ATP generated through respiration (Table 3). The P/O ratios were also estimated from the electron flux to the respiratory chain and the estimated ATP generation through respiration. Assimilatory NADH generation was estimated from the anaerobic glycerol production rate, assuming that NADH generation in biosynthetic reactions was constant per g CDW^-1 ^in all conditions. The generation of electron donors, NADH and FADH_2_, in central carbon metabolism was determined from the net flux data. As NADH and FADH_2 _are energetically equivalent in yeast, the estimated total electron flux to the respiratory chain was calculated from the summed generation of electron donors. The two estimates for P/O ratios were close to one in all the conditions.

### Transcriptional regulation of metabolic enzymes

Results from Transcript analysis with the aid of Affinity Capture (TRAC) of *S. cerevisiae *in the different oxygenation conditions are presented in Wiebe *et al. *(2008) [32]. Genes encoding enzymes of central carbon metabolism were mapped to the corresponding fluxes in the metabolic network model using the *Saccharomyces *Genome Database [47] and Pearson correlation coefficients were calculated between the transcription levels of genes encoding metabolic enzymes and the fluxes through the enzymes in the different conditions. Positive correlation (> 0.60) between the transcriptional levels [32] and the corresponding fluxes in the different oxygenation conditions was found only in the respirative pathway, i.e. in pyruvate dehydrogenase and in the TCA cycle (Additional file 3).

## Discussion

The dependence of the intracellular metabolic flux distribution of *S. cerevisiae *CEN.PK113-1A on the external oxygen availability was studied in glucose-limited chemostats under five different oxygenation conditions with ^13^C-labelling. ^13^C-labelling was utilised to obtain ratios of intracellular fluxes at the metabolic branching points [23,43]. The flux ratio constraints were included in the MFA systems to solve the metabolic flux distributions [24]. The redox cofactors NADH and NADPH were not included in the metabolite mass balancing in ^13^C-MFA so that the intracellular flux distributions could be reliably solved, despite the lack of precise information on the cofactor specificities and the relative activities of different isoenzymes for conditions in which redox balancing is an important determinant of cell physiology, in particular metabolic fluxes. The dilution rate in the chemostat cultivations, 0.10 h^-1^, was well below the μ_max _observed for the equivalent strain CEN.PK113-7D: 0.41 h^-1 ^and 0.30 h^-1 ^in aerobic and anaerobic conditions [15], respectively, and significantly lower than the critical dilution rate 0.27 h^-1^, at which the metabolism of *S. cerevisiae *(CEN.PK122) has been reported to shift from fully respirative to respiro-fermentative in aerobic chemostat cultures [42]. The entirely respirative metabolism of *S. cerevisiae *under fully aerobic conditions was further confirmed by the absence of ethanol and other fermentation products in the culture supernatant and approximately the same specific rates of O_2 _consumption and CO_2 _production (Table 1). The controlled continuous culture conditions ensured that the metabolic effects observed under conditions of restricted respiration in the current study stemmed solely from the reduced availability of oxygen, rather than from exceeding the respiratory capacity, which has been observed to result in overflow metabolism, in aerobic alcoholic fermentation at high specific growth rate [17,18].

The switch from entirely respirative metabolism to respiro-fermentative metabolism was observed in conditions of 2.8% oxygen in the chemostat inlet gas. However, in 2.8% oxygen the respirative pathways still carried most of the carbon fluxes. When the oxygen provision was further restricted to 1.0%, thus reducing the potential of respirative ATP production, flux through the fermentative pathway increased. Since mitochondrial respiration is a significantly more efficient means to produce ATP than substrate level phosphorylations, even in only 0.5% oxygen a significant fraction (25%) of ATP was produced through respiration.

Major redistributions of carbon fluxes were observed between the different oxygenation conditions, particularly at the pyruvate branch point where the metabolism branches to three pathways. The respirative and the fermentative pathways branch out from pyruvate through the enzymes pyruvate dehydrogenase and pyruvate decarboxylase, respectively. Pyruvate decarboxylase has been found to be essential for growth on glucose in *S. cerevisiae *because of the assimilatory role of the pathway in generation of cytosolic acetyl-CoA. It is therefore also expressed during respiratory growth [48]. Wiebe *et al. *(2008) observed decreased expression of the pyruvate decarboxylase *PDC1 *gene in low oxygenation [32] although the flux redistribution at the pyruvate branch point demonstrated that higher flux was directed through pyruvate decarboxylase in low than in high external oxygen, suggesting that post-transcriptional regulation is important for pyruvate decarboxylase. In 1.0% oxygen the fermentative flux through pyruvate decarboxylase became the main carbon flux from the pyruvate branch point.

Under glucose repression the respiratory pathway enzymes are severely down-regulated [17] whereas under low external oxygen availability the respiratory chain is functional but the terminal electron acceptor, oxygen, is limiting. The electron transport chain may even be optimized for low oxygen conditions by oxygen dependent modification of the terminal electron acceptor COX subunits Cox5a and Cox5b via transcriptional regulation [49]. The genes encoding TCA cycle enzymes are down-regulated in low oxygenation [32]. The carbon fluxes in the TCA cycle were also lower in lower oxygenation. In anaerobic conditions the TCA cycle operated as a branched pathway, as previously observed by Fiaux *et al. *(2003) [20] and in aerobic glucose repressed batch cultures by Gombert *et al. *(2001) [19]. On the contrary Maaheimo *et al. *(2001) [34] observed cyclic operation of the TCA cycle in aerobic batch cultures and branched operation only in anaerobic batch cultures. In mammals pyruvate dehydrogenase can be regulated via HIF1 mediated phosphorylation to reduce the flux to the TCA cycle under restricted respiration [50]. However, the activity of the *S. cerevisiae *pyruvate dehydrogenase enzyme has not been found to be regulated by phosphorylation [51].

The third flux branching from pyruvate, the anaplerotic flux, through pyruvate carboxylase, replaces the carbons lost from the TCA cycle to biosynthesis. An increase in the anaplerotic flux can be expected when the ratio of the carbon flow to biosynthesis, relative to the respirative carbon flux through the TCA cycle, is increased. When the respiration rate was reduced by the reduced availability of external oxygen while the growth rate was kept constant, the respirative carbon flux was decreased whereas the specific carbon flux to biosynthesis remained the same. In 0.5% oxygen the respirative carbon flux was still over 60% of the net carbon flux to the TCA cycle whereas in anaerobic conditions there was no respirative carbon flux and the anaplerotic flux was the only source of Oaa_mit_. Frick and Wittmann (2005) observed considerably increased anaplerotic fluxes in *S. cerevisiae *at high growth rates (D = 0.30, 0.40 h^-1^) in aerobic chemostats compared to low growth rate (D = 0.15 h^-1^) and the increases in the anaplerotic fluxes were accompanied by high malic enzyme fluxes [18]. High contribution of a malic enzyme flux has also been observed in aerobic glucose-repressed batch cultures [34]. In this study the highest, but still low, malic enzyme fluxes were observed in the more oxygenised conditions while the absolute anaplerotic flux remained on fairly constant level and only the ratio of anaplerosis to the TCA cycle flux was increased when oxygen concentration was reduced. Thus a high ratio of anaplerotic flux to the TCA cycle flux is associated with respiro-fermentative and anaerobic metabolism, but high absolute anaplerotic and malic enzyme fluxes with high specific growth rate and/or overflow metabolism. Overflow metabolism was not observed as a result of decreased respiratory rate achieved by reduced oxygen provision.

In fully aerobic conditions *S. cerevisiae *regenerates NAD^+ ^mainly through respiration. When limited oxygen availability restricts respiration, cells are forced to use other means for regeneration of NAD^+^and mitochondrial NADH needs to be transported to the cytosol for reoxidisation. For the transport of NADH, mitochondrial alcohol dehydrogenase, encoded by *ADH3*, provides a probable redox shuttle [6,52]. *S. cerevisiae *oxidises the surplus NADH by producing glycerol as a redox sink. In this study, carbon loss to glycerol was observed only in anaerobic conditions, as expected. Based on the theoretical amount of assimilatory NADH synthesised in anaerobic conditions, 11 mmol g biomass^-1 ^at a growth rate 0.1 h^-1 ^[10], which was consistent with the anaerobic glycerol production rate observed in this study (1.2 mmol g biomas^-1 ^h^-1 ^[32], no net glycerol production should occur for oxygen uptake rates of 0.55 mmol O_2 _g biomass^-1 ^h^-1 ^or higher [53]. The oxygen uptake rate measured in the lowest oxygen concentration provided in this study, 0.5% O_2_, was 1.2 mmol O_2 _g biomass^-1 ^h^-1^, which is twice the rate which would be sufficient for maintaining the cytosolic NADH balance with the external NADH dehydrogenases and mitochondrial respiration [6].

The main mechanisms in *S. cerevisiae *for mitochondrial reoxidation of cytosolic NADH are the external NADH dehydrogenases (Nde1p and Nde2p) but the glycerol-3-phosphate shuttle is also known to be active [6,7]. The anaerobic flux distribution observed was clearly different from all the other flux distributions since respiration could maintain the NADH/NAD^+ ^ratio in all the aerobic conditions. Weusthuis *et al. *(1994) indicated that yeasts could optimise their function for redox balancing so that available oxygen would primarily be used to maintain the redox balance, thus avoiding carbon loss to glycerol [8]. The indication has been supported by MFA modelling results of *S. cerevisiae *metabolism in low oxygen conditions [9]. Respiratory functions couple energy generation in terms of ATP with the redox balance. Since the redox cofactor NADH is one of the hub metabolites in the organism-wide network of metabolic reactions [29], the regulation of redox homeostasis encompasses all the metabolic pathways.

In this study P/O ratios were estimated in two different ways: from OURs and from the flux of electrons to the respiratory chain. The two different estimates were consistent with each other and close to one in all conditions. The effective P/O ratio has previously been estimated to be close to one in respiratory, carbon-limited cultures [54] and an increase in the effective P/O ratio in decreased respiratory fluxes has been observed in isolated mitochondria and in spheroplasts [55-57]. An ability to adjust the P/O ratio has been discussed as providing an important means to control ATP synthesis in cells to adapt to changes in energy demands [56]. In this study no significant increase in the P/O ratio was observed with decreasing respiratory fluxes.

The PPP provides precursors for biosynthesis and reductive power in the form of NADPH. The relative flux to the PPP appeared to be mainly determined by the NADPH requirement for biomass synthesis in the different oxygenation conditions. It has been stated that the flux through the PPP depends on the NADP^+^/NADPH ratio in the cell and additionally on the MgATP^2- ^pool that inhibits glucose-6-phosphate dehydrogenase, an enzyme in the oxidative branch of the PPP, allowing dynamic regulation of the relative PPP flux [58]. The dependence of the relative PPP flux on growth rate and biomass yield has been observed [18]. The relative PPP flux contributions to PEP observed with METAFoR analysis of glucose repressed cells in aerobic batch cultures [34] are essentially the same as observed in this study in response to the lowest oxygen provision and anaerobic conditions. NADPH production of the oxidative PPP in the aerobic cultivations was approximately 6 mmol g biomass^-1 ^in this study, assuming the maximum relative PPP flux, while approximately 9 mmol g biomass^-1 ^would be needed for reducing power in the form of NADPH for biomass production of yeast growing on glucose with ammonium as the nitrogen source [59]. Thus one third of the NADPH required must have been produced in pathways other than the PPP. The isocitrate dehydrogenase reaction of the TCA cycle is assumed to be another main contributor to the production of NADPH [59]. However, NADPH is also known to be an important factor in oxidative damage prevention [60] and therefore the NADPH requirement may have been lower in the lower oxygenation conditions.

The changes in metabolic flux distribution observed in the series of different oxygenation conditions were positively correlated with the transcriptional changes of the genes encoding the flux carrying metabolic enzymes [32] only for pyruvate dehydrogenase and the TCA cycle. Glycolytic flux, in particular, showed a large increase as oxygenation was reduced, in contrast to the expression levels of some of the corresponding genes [32]. However, no extensive correlation between changes in transcription and the flux distribution in aerobic and anaerobic chemostat cultures of *S. cerevisiae *has been previously observed [61,62] and the glycolytic enzymes have recently been stated to be post-transcriptionally regulated [37,63]. In contrast, some transcriptional regulation of metabolism has been found to correlate with the glycolytic rate in batch cultures of *S. cerevisiae *strains displaying glucose uptake rates between 3.5 mmol g^-1 ^h^-1 ^and 15.8 mmol g^-1 ^h^-1 ^by Elbing *et al. *(2004) [64]. In this study, even though the specific glucose uptake rates in chemostats varied between 0.9 mmol g^-1 ^h^-1 ^and 6.6 mmol g^-1 ^h^-1 ^[32] there was no correlation with the transcriptional level of the glycolytic genes which were studied. However, in the work by Elbing *et al. *(2004), how the glycolytic rate was sensed to trigger transcriptional changes was not resolved [64]. As extensive oxygen dependent redistributions of fluxes were observed in central carbon metabolism in this work, the oxygen-dependent regulation of the fluxes in *S. cerevisiae *appears to lie mainly at the post-transcriptional level of the cell's regulatory system. However, it should be kept in mind that the oxygen dependent flux distributions of *S. cerevisiae *reflect not only the direct oxygen sensing regulatory mechanisms, but rather the ultimate response of the whole interactive multi-level regulatory system.

## Conclusion

In this study the quantification of the flux distributions of *S. cerevisiae *in response to different oxygenation conditions with ^13^C-MFA showed that the fluxes were redistributed not only between the cells grown in the fully aerobic conditions, in conditions of reduced oxygen provision and in anaerobic conditions but also for cells grown with 2.8%, 1.0% and 0.5% oxygen. Although the cellular metabolism was respiro-fermentative in each of these low oxygen conditions, the actual amount of oxygen available resulted in different contributions through respirative and fermentative pathways. The flux distribution at the pyruvate branch point, leading to respirative and fermentative pathways and to anaplerotic flux replenishing the TCA cycle, was particularly responsive to the level of reduction in oxygen provision. The oxygen-dependent regulation of the flux distribution in central carbon metabolism of *S. cerevisiae *appeared to lie mainly at the post-transcriptional level of the cell's regulatory system. Respirative pathway flux decreased progressively in reduced oxygenation conditions where the availability of terminal electron acceptor limited the respiratory rate. However, respiratory energy generation, being very efficient, provided a large fraction of the ATP required even in low oxygen conditions.

## Methods

### Strain and medium

*Saccharomyces cerevisiae *CEN.PK113-1A (*MATα*, *URA3*, *HIS3*, *LEU2*, *TRP1*, *MAL2-8c*, *SUC2*) was kindly provided by Dr. P. Kötter (Institut für Mikrobiologie, J.W. Goethe Universität Frankfurt, Germany) [65] and stored in glycerol (30% v/v) at -80°C [32].

Yeast were grown in defined minimal medium [66], with 10 g glucose l^-1 ^as carbon source, and supplemented with 10 mg ergosterol l^-1 ^and 420 mg Tween 80 l^-1 ^(a source of oleic acid). BDH silicone antifoam (0.5 ml l^-1^) was used to prevent foam production in the cultures [32].

### Culture conditions

Cells were grown in 0.8 to 1 l medium in Biostat CT (2.5 l working volume) bioreactors. Cultures were inoculated to an initial OD_600 _of approximately 0.5 and maintained as batch cultures for 6 to 9 h, after which continuous medium feed was started while the cells were still growing exponentially. Chemostat cultures were maintained at D = 0.10 ± 0.02 h^-1^, pH 5.0, 30°C, with 1.5 volume gas [volume culture]^-1 ^min^-1 ^(vvm). Chemostat cultivations were performed with five different oxygenation conditions: 20.9%, 2.8%, 1.0%, 0.5% and 0.0% oxygen of the chemostat inlet gas. For cultures which received less than 20.9% O_2 _in the gas stream, O_2 _was replaced with the equivalent volume of N_2_, so that total gas flow was maintained constant for all experiments. The N_2 _gas used was 99.999% pure. Gas concentration (CO_2_, O_2_, N_2 _and Ar) was analysed continuously in an Omnistar quadrupole mass spectrometer (Balzers AG, Liechenstein), calibrated with 3% CO_2 _in Ar. ^13^C labelled CO_2 _was taken into account in the determination of CERs during feeding with^13^C glucose.

To achieve anaerobic conditions in the chemostat only Marprene tubing with very low oxygen permeability was used to connect the vessels. The medium reservoir was continually flushed with N_2 _to prevent additional O_2 _being added by diffusion into the medium in the anaerobic and low oxygen cultures. The k_L_a (overall oxygen transfer coefficient) for the bioreactor in the cultivation conditions was 0.035–0.039s^-1 ^(in pure water). The DOT was 83% in cultures receiving 20.9% O_2 _and 0% in all the other cultures [32]. It should be noted that, based on Henry's law, the amount of oxygen able to dissolve into the medium is determined by the partial pressure of oxygen in the inlet gas, and that in oxygen-limited conditions (i.e. cultures receiving 2.8, 1.0 or 0.5% oxygen) the yeast were able to utilise all the oxygen which was able to dissolve into the medium. Oxygen was continually dissolving and continually being removed. Thus the oxygen available to the yeast was directly determined by the concentration of oxygen in the inlet gas while the measurable DOT remained zero, as also indicated by the OURs. Dissolved oxygen was measured with a Mettler Toledo InPro(R) 6000 series polarographic dissolved oxygen probe.

The culture conditions and biomass determination and chemical and metabolite analyses are described in more detail by Wiebe *et al. *(2008) [32]. The rate of ethanol loss through evaporation was estimated, based on initial measurements for 1 l cultures at 30°C, 15 vvm aeration and 800 rpm agitation and assuming that the evaporation rate would be constant in chemostat cultures under these conditions.

### Biosynthetically directed fractional (BDF) ^13^C-labelling

^13^C-labelling experiments were performed in at least two replicate cultures under each oxygenation condition. After reaching a metabolic steady state, as determined by constant physiological parameters including biomass production, carbon dioxide evolution and oxygen uptake rates (CER and OUR), alkali utilisation, and subsequently confirmed by the observation of constant extracellular and intracellular metabolites and gene transcription, 10% of the carbon source in the medium was replaced with [U-^13^C]glucose (Isotec, 99 atom% ^13^C). During steady state growth the active pathways in the cells will determine how the yeast biomass becomes ^13^C-labelled. After approximately 1.5 residence times biomass samples, 50 ml of culture broth, corresponding to 0.27 to 0.05 g CDW, were harvested by centrifugation. The cell pellets were suspended into 10 ml of 6 M HCl and the biomass was hydrolysed in sealed glass tubes at 110°C for 22 h. The suspensions were dried and dissolved in H_2_O for filtration through 0.2 μm filters. The filtrates were vacuum-dried and dissolved in D_2_O for NMR experiments. The pH of the samples was below 1 due to residual HCl.

As described previously [20,23,34,43,67-69], the calculation of metabolic flux ratios when using fractional ^13^C-labelling of amino acids is based on the assumption that both a metabolic and an isotopomeric steady state exist. To establish a cost-effective protocol for a larger number of ^13^C-labelling experiments, ^13^C-labelled substrate was fed to a chemostat operating in a metabolic steady state for the duration of 1.5 volume changes [20,67] before harvesting the biomass. The fraction of unlabelled biomass produced prior to the start of ^13^C-labelled medium supply was calculated following simple wash-out kinetics [69].

### NMR spectroscopy

^13^C-HSQC nuclear magnetic resonance (NMR) spectra were acquired at 40°C on a Varian Inova spectrometer operating at a ^1^H-resonance frequency of 600 MHz essentially as described [43]. For each sample two spectra were acquired focusing on the aliphatic and aromatic regions. For the aliphatic spectra, a matrix of 1024 × 1500 (f2 × f1) complex data points was acquired and zero-filled to 4096 complex data points in f1. The spectral widths were 6000 Hz and 5100 Hz in the ^1^H- and ^13^C-dimensions, respectively. The narrow spectral width in the ^13^C-dimension leads back-folding of part of the signals to the empty regions of the spectrum. For the aromatic region, a matrix of 1024 × 800 complex data points was acquired and zero-filled to 2048 complex data points in f1. The spectral widths for the aromatic spectra were 6000 Hz and 2815 Hz in the ^1^H- and ^13^C-dimensions, respectively. All spectra were weighted with a cosine function in both dimensions prior to the Fourier transformation. The spectra were processed using the standard Varian spectrometer software VNMR (version 6.1, C).

### METAFoR analysis

Metabolic flux ratio (METAFoR) analysis was done based on the compartmentalized metabolic model of *S. cerevisiae *central carbon metabolism formulated by Maaheimo and co-workers (2001) [34]. The software FCAL (R.W. Glaser; FCAL 2.3.1) [23] was used for the integration of ^13^C-scalar fine structures of proteinogenic amino acid carbon signals in the ^13^C-HSQC NMR spectra and the calculation of relative abundances of intact carbon fragments originating from a single source molecule of glucose. The nomenclature used here for the intact carbon fragments, fragmentomers, has been described previously [43]. Briefly, *f*^(1) ^represents the fraction of molecules in which the observed carbon atom and the neighbouring carbons originate from different source molecules of glucose, and *f*^(2) ^the fraction of molecules in which the observed carbon atom and one of the two neighbouring atoms originate from the same source molecule of glucose, and *f*^(3) ^the fraction of molecules in which the observed carbon atom and both the neighbouring carbons originate from the same source molecule of glucose. If the observed carbon exhibits significantly different ^13^C-^13^C scalar coupling constants with the neighbouring carbons, *f*^(2)^and *f*^(2*) ^can be distinguished. The fraction of molecules with a conserved bond between the observed carbon atom and the neighbouring carbon with the smaller coupling is represented by *f*^(2)^. *f*^(2*) ^then denotes for fraction of molecules where the bond is conserved between the observed carbon and the neighbouring carbon with the larger coupling. If the observed carbon is located at the end of a carbon chain, *f*^(1) ^and *f*^(2) ^fragmentomers can be observed indicating the conservation of the terminal two carbon fragment of the molecule.

Fragmentomer information obtained from proteinogenic amino acids can be traced back to the metabolic intermediates in central carbon metabolism since the carbon backbones of eight intermediates are conserved in amino acid synthesis pathways [34]. Mass balance equations of specific carbon fragments of the metabolic intermediates were formulated from the propagated fragmentomer information for junctions in central carbon metabolism.

Since glycolysis and the pentose phosphate pathway (PPP) are completely located in the cytosol, the upper bound for the fraction of Pep from the PPP was calculated as for prokaryotic cells and as described by Maaheimo and co-workers (2001) [34]. The fraction of Pep originating from phosphoenolpyruvate carboxykinase activity, denoted by *X*_*PEPck*_, was calculated from the ratio of the fraction of Pep molecules containing an intact C1–C2 fragment and a cleaved bond between C2 and C3 and the fraction of Oaa_cyt _molecules containing the equivalent fragments (Equation 1). These fragments cannot originate from glycolysis or from the PPP [34]. Phe-Cα, Tyr-Cα and Asp-Cα, Thr-Cα can be traced back to the C2 of Pep and Oaa_cyt_, respectively, as the amino acid synthesis pathways from metabolic intermediates are known [34] (Equation 1).

(1) *X*_*PEPck *_= *Pep*_10/*Oaa*_*cyt*__10*x *= [*f *^(2*)^]{*phe*, *Tyr*-*Cα*}/[*f *^(2*)^]{*Asp*, *Thr*-*Cα *}

where *Pep*_10 denotes for the fraction of Pep molecules that possess an intact C1–C2 bond and cleaved C2–C3 bond and *Oaa*_*cyt*__10*x *denotes for the fraction of Oaa_cyt _molecules that possess an intact C1–C2 bond, a cleaved C2–C3 bond and either intact or cleaved C3–C4 bond.

The Oaa_mit _molecules originating from Oga through the TCA cycle possess cleaved C2–C3 bonds. The fraction of Oaa_mit _originating from transport over the mitochondrial membrane from Oaa_cyt _was solved from a mass balance of intact C2–C3 fragments in Oaa_mit_. The conserved connectivity of the C2–C3 fragment in Oaa_mit _can be propagated back from Glu-Cα and Pro-Cα carbons that represent the C2 carbon in Oga, since the C2–C3 fragment of Oaa_mit _is conserved in the TCA cycle as the C2–C3 fragment of Oga. The fraction of Oaa_mit _from Oaa_cyt_, denoted by *X*_*Oaa-transport*_, was calculated as a ratio of intact C2–C3 fragments in Oga and Oaa_cyt _(Equation 2).

(2) $$\begin{array}{l} X_{Oaa-transport}=Oga\_x1xx/Oaa_{cyt}\_x1x\\ =\left(\left[f^{\left(2\right)}+f^{\left(3\right)}\right]\left\{Glu,\mathrm{Pr}o-C\alpha \right\}\right)/\left(\left[f^{\left(2\right)}+f^{\left(3\right)}\right]\left\{Asp,Thr-C\alpha ,Asp-C\beta \right\}\right) \end{array}$$

The fraction of Oaa_cyt _originating from Pyr_cyt_, denoted by $X_{Oaa_{cyt}\_from\_Pyr_{cyt}}$, was solved from the mass balance of intact C2–C3 fragments (Equation 3). Since the flux from Pep to Pyr_cyt _through phosphoenolpyruvate kinase and further through pyruvate carboxylase to Oaa_cyt _can be assumed to be irreversible in the experimental conditions used here, the C2–C3 fragments of Pep were used in the mass balance equations. The conserved connectivity of the C2–C3 fragment in Pyr_cyt _can be observed from Phe-Cα and Tyr-Cα that represent the C2 carbon of Pep (Equation 3).

(3) XOaacyt_from_Pyrcyt=(Oaacyt_x1x−Oga_x1xx)/(Pep_x1−Oga_x1xx)=[f(2)+f(3)]{Asp,Thr−Cα,Asp−Cβ}−[f(2)+f(3)]{Glu,Pr⁡o−Cα}[f(2)+f(3)]{Phe,Tyr−Cα,Cβ}−[f(2)+f(3)]{Glu,Pr⁡o−Cα}

The upper and lower bounds for Pyr_mit _originating from the malic enzyme reaction, denoted by *X*_*MAE_ub *_and *X*_*MAE_lb *_respectively, were calculated from a mass balance of intact C2–C3 fragments of Pyr_mit _(Equations 4 and 5). The upper and lower bounds were obtained from the assumption that the substrate fragment for malic enzyme has an equally conserved connectivity as Oga and Oaa_mit_. The intact fragments in Oaa_mit _were obtained from the intact fragments in Oga since the C2–C3–C4 fragment of Oaa_mit _is conserved in the TCA cycle in synthesis of Oga. The intact fragments in biosynthetic precursor Oga were deduced from the *f*-values of Glu and Pro carbons (Equations 4 and 5).

(4) $$\begin{array}{l} X_{MAE\_ub}=\left(Pep\_x1-Pyr_{mit}\_x1\right)/\left(Pep\_x1-Oga\_x1xx\right)\\ =\frac{\left[f^{\left(2\right)}+f^{\left(3\right)}\right]\left\{Phe,Tyr-C\alpha ,C\beta \right\}-\left[f^{\left(2\right)}+f^{\left(3\right)}\right]\left\{Ala-C\alpha ,C\beta \right\}}{\left[f^{\left(2\right)}+f^{\left(3\right)}\right]\left\{Phe,Tyr-C\alpha ,C\beta \right\}-\left[f^{\left(2\right)}+f^{\left(3\right)}\right]\left\{Glu,\mathrm{Pr}o-C\alpha \right\}} \end{array}$$

(5) $$\begin{array}{l} X_{MAE\_lb}=\left(Pep\_x1-Pyr_{mit}\_x1\right)/Pep\_x1\\ =\frac{\left[f^{\left(2\right)}+f^{\left(3\right)}\right]\left\{Phe,Tyr-C\alpha ,C\beta \right\}-\left[f^{\left(2\right)}+f^{\left(3\right)}\right]\left\{Ala-C\alpha ,C\beta \right\}}{\left[f^{\left(2\right)}+f^{\left(3\right)}\right]\left\{Phe,Tyr-C\alpha ,C\beta \right\}} \end{array}$$

### ^13^C-MFA

Metabolic flux analysis (MFA) was used to determine intracellular metabolic fluxes, with METAFoR analysis providing additional experimental constraints to solve the MFA system [24]. A stoichiometric model of central carbon metabolism of *S. cerevisiae *was formulated, based on the model used in the METAFoR analysis [34] (Additional file 4). The model included the glycolytic and the pentose phosphate pathways, the TCA cycle and the fermentative pathways, production of glycerol and anabolic fluxes from metabolic intermediates to biosynthesis. The glyoxylate cycle was omitted from the model since the METAFoR data showed that the pathway was inactive. The labelling pattern of succinate that would have originated from the glyoxylate cycle was calculated from Asp and Lys fragmentomers representing the labelling patterns of Oaa_cyt _and AcCoA_cyt _respectively. No trace of influx of succinate originating from the Glyoxylate cycle to the TCA cycle was observed since the labelling pattern of Oga derived from Glu fragmentomers was fully explained by the TCA carbon flux. Separate pools of Pyr, AcCoA and Oaa in the two cellular compartments, cytoplasm and mitochondria, were included in the model. Mal was lumped in the same pool with Oaa_mit_. Also the pentose phosphates formed a single pool and the triose phosphates were combined in the pools of G3P and Pep. DHAP, the precursor for glycerol synthesis, was also combined with the G3P pool. TCA cycle metabolites were represented by the pools of citrate, Oga and Oaa_mit_. Scrambling of ^13^C-labels in the symmetric molecules succinate and fumarate was taken into account. The transport of Pyr and Oaa across the mitochondrial membrane were included in the model but the transport of AcCoA, the final step of the cytosolic PDH bypass, was omitted since exogenous carnitine would be required for the carnitine shuttle to be active [70-72], and carnitine was not provided in the medium. In addition carnitine acetyltransferase activity has not been detected in *S. cerevisiae *grown in anaerobic chemostats at 0.1 h^-1 ^[35]. Since acetaldehyde can freely diffuse across the mitochondrial membrane and acetaldehyde dehydrogenase (EC 1.2.1.3) and AcCoA synthetase (EC 6.2.1.1) enzymes have both been isolated in the mitochodrial proteome [73], PDH bypass could also be partially located in mitochondria and contribute directly to the formation of AcCoA_mit_. In absence of fluxes inducing significantly dissimilar labelling patterns to the C2–C3 fragments of Pyr_cyt _and Pyr_mit _i.e. in conditions of low malic enzyme fluxes as observed in this study, ^13^C-labelling cannot solely reveal the possible contribution of PDH bypass pathways to the carbon flux to mitochondria. However, in the cultivations performed, the expression of *ACS1 *encoding the mitochondrial AcCoA synthetase, essential for the contribution of mitochondrial PDH bypass to the formation of AcCoA_mit_, was negligible and the expression of *ACS2 *encoding the cytosolic isoenzyme was substantially higher [32]. Thus, the mitochondrial PDH bypass was not included in the model.

The metabolic fluxes were modelled as net fluxes so that a net flux in the forward direction was assigned with a positive value and a net flux in the reverse direction was assigned with a negative value. As an exception, the transport of Oaa across the mitochondrial membrane was modelled as two one-directional transport reactions. In *S. cerevisiae *the transport of OAA across the mitochondrial membrane can occur via mitochondrial Oaa transporter OAC1 facilitated transport [74].

The stoichiometric model for experiments in 20.9%, 2.8% and 1.0% oxygen conditions consisted of 38 reactions coupling 34 metabolites including duplicated extracellular metabolites and uptake and production fluxes, Figure 4. The 14 fluxes across the system boundary included glucose uptake and excretion fluxes of ethanol, acetate and glycerol and the fluxes of the metabolic precursors to macromolecule synthesis for biomass production. The METAFoR results were used to identify inactive reactions, to constrain the stoichiometric models for the experiments with 0.5% and 0.0% oxygen by omitting inactive fluxes. The stoichiometric model for experiments in 0.5% oxygen consisted of 37 reactions, coupling 34 metabolites and excluding the malic enzyme activity from the first model of the network of active reactions. The compartmentalization of central carbon metabolism in anaerobic conditions is evident from the vital anabolic role of mitochondria in the absence of oxygen [75]. However, in completely anaerobic conditions only the net transport of Oaa across the mitochondrial membrane is resolvable and the activities of PEPck and malic enzyme reactions cannot be quantified. Since, according to the METAFoR analysis, the PEPck reaction showed only slight activity in the other conditions studied and its activity decreased as the oxygen provided was reduced, it was omitted from the anaerobic stoichiometric model. *MAE1 *has been shown to be induced in anaerobic conditions and its possible role in provision of NADPH in mitochondria in anaerobic conditions has been discussed [76]. However, the malic enzyme reaction also showed only slight activity in all the conditions where quantification was possible and had its lowest activity in 0.5% oxygen. Thus, the malic enzyme reaction was omitted from the anaerobic model. Under anaerobic conditions the stoichiometric model of the active pathways consisted of 34 reactions and 34 metabolites.

After including the measured uptake and excretion rates and the rates of metabolic precursor depletion to biomass synthesis, as determined from the composition of *S. cerevisiae *biomass previously reported [19], in the models, the linear equation systems remained underdetermined. The composition of *S. cerevisiae *biomass was assumed to be the same in all the conditions studied, since the biomass composition in the two extreme conditions, i.e. in fully aerobic and in anaerobic conditions, has been experimentally shown to be essentially the same [19,35]. Solvable systems were obtained by further constraining the MFA systems with one to six linearly independent constraints, depending on the structure of the network of active reactions from the METAFoR analysis as described by Fischer and co-workers (2004) [24]. Using the constraints from the METAFoR analysis, it was not necessary to include redox cofactor mass balances in the mass balance constraints in ^13^C MFA. Cofactor mass balances are sources of errors since the correct balancing requires detailed knowledge of the relative activities of different isoenzymes and the enzyme cofactor specificities on a cell wide scale. The mass balances of the metabolites were formulated as a linear equation system as described in [24] (Equation 6):

(6) *N*_*i*_*x *- *b *= *R*_*m*_

where *N*_*i *_is the stoichiometric matrix of the active network *i *determined from the METAFoR analysis fragmentomer data, *x *is the flux distribution vector, *b *is the vector of the measured extracellular fluxes and *R*_*m *_is the vector of the residuals of metabolite mass balances.

The flux ratio equations were set up according to the METAFoR analysis for the reactions in the stoichiometric models of the central carbon metabolism of *S. cerevisiae *(Equations 7 to 11, the reaction numbers are defined in Figure 4). Depending on the structure of the network of active reactions the flux ratio equations included one to six of the following (Equations 7 to 11):

the fraction of Pep from PPP assuming a maximal contribution of PPP

(7) $$fr1=\frac{x_{5}+3x_{6}+2x_{7}}{x_{5}+2x_{4}+x_{6}}$$

the fraction of Pep originating from Oaa_cyt_, *X*_*PEPck*_:

(8) $$fr2=\frac{x_{15}}{x_{15}+x_{8}}$$

the fraction of Oaa_mit _originating from Oaa_cyt_, *X*_*Oaa-transport*_:

(9) $$fr3=\frac{x_{21}}{x_{21}+x_{13}}$$

the fraction of Oaa_cyt _originating from Pyr_cyt_, $X_{Oaa_{cyt}\_from\_Pyr_{cyt}}$:

(10) $$fr4=\frac{x_{16}}{x_{16}+x_{22}}$$

the upper and lower bounds for Pyr_mit _originating from the malic enzyme reaction, *X*_*MAE_ub *_and *X*_*MAE_lb*_:

(11) $$fr5\leq \frac{x_{14}}{x_{14}+x_{9}}\leq fr6$$

The following linear constraint equations were obtained from the flux ratio equations and included to the MFA systems to solve the metabolite mass balances (Equations 12 to 17):

(12) *x*_5 _+ 3*x*_6 _+ 2*x*_7 _- *fr*1(*x*_5 _+ 2*x*_4 _+ *x*_6_) = 0

(13) *x*_15_*- fr *2(*x*_15_*+ x*_8_) = 0

(14) *x_21_*-*fr*3(*x*_21 _+ *x*_13_) = 0

(15) *x*_16 _- *fr*4(*x_16 _*+ *x*_22_) = 0

(16) *x*_14 _- *fr*5(*x*_14 _+ *x*_9_) = 0

(17) *fr*6(*x*_14 _+ *x*_9_)-*x*_14 _= 0

Irreversibility was assumed for the intracellular fluxes *x*_3_, *x*_4_, *x*_8_, *x*_9_, *x*_10_, *x*_11_, *x*_12_, *x*_13_, *x*_14_, *x*_15_, *x*_16_, *x*_21_, *x*_22_, *x*_23_, *x*_24_, for extracellular fluxes *x*_25_, *x*_26_, *x*_27_, *x*_28_, and for the depletion of precursors to biosynthetic reactions and thus, only positive values were allowed for the fluxes. The minimization of the sum of the weighted square residuals of the metabolite mass balances was done using the Matlab function *fmincon*. The extracellular metabolite mass balances were assigned weights according to the experimental measurement error estimates. The biomass precursor metabolite mass balances were assigned ten-fold larger weights, relative to their stoichiometric coefficients in the biomass composition, since the biomass composition was assumed constant in all the conditions studied [2]. The flux ratio constraints were included as strict constraints. The optimization was started 10000 times from random initial values to evaluate the uniqueness of the optimal flux distribution. The sensitivity of the flux distribution solutions to the noise in the flux ratio data and in the extracellular flux data was studied by Monte Carlo-simulations [77]. Additive normally distributed noise with zero mean and experimentally determined variances of the flux ratios and the extracellular fluxes was sampled to the flux ratios and to the extracellular flux data, separately and simultaneously. A flux distribution was solved for each of the 100 sets of input data from 12 random initial flux distributions. Confidence intervals (95%) for the fluxes were determined.

## Abbreviations

AcCoA: acetyl coenzyme A; AcO: acetaldehyde; CDW: cell dry weight; Cit: citrate; DHAP: dihydroxyacetone phosphate; E4P: erythrose 4-phosphate; F6P: fructose 6-phosphate; G3P: glyceraldehyde 3-phosphate; G6P: glucose 6-phosphate; HSQC: heteronuclear single quantum coherence; Mal: malate; METAFoR: metabolic flux ratio; Oaa: oxaloacetate; Oaa_cyt_: cytosolic oxaloacetate; Oaa_mit: _mitochondrial oxaloacetate; Oga: 2-oxoglutaric acid; Pep: phosphoenolpyruvate; PEPck: phosphoenolpyruvate carboxykinase; PPP: pentose phosphate pathway; Pyr: pyruvate; Pyr_cyt: _cytosolic pyruvate; Pyr_mit: _mitochondrial pyruvate; P5P: pentose 5-phosphate; S7P: sedoheptulose 7-phosphate; TCA: tricarboxylic acid.

## Authors' contributions

PJ participated in the design of the study, performed chemostat cultivations, carried out the NMR experiments, designed and performed the modeling and the computational work and drafted the manuscript, ER and MT participated in the design of the study and performed chemostat cultivations, AH and AT performed chemostat cultivations, MW participated in the design of the study, performed chemostat cultivations and the calculations of the physiological parameters and revised the language of the manuscript, LR and MP participated in the design of the study, HM participated in the design of the study and the NMR experiments and helped to draft the manuscript. All authors read and approved the final manuscript.

## Supplementary Material

Additional file 1 **Relative abundances of intact carbon fragments in proteinogenic amino acids**. Relative abundances of intact C2 and C3 fragments (*f*-values) in proteinogenic amino acids describing the conservation of carbon chain fragments in the metabolism of *S. cerevisiae *CEN.PK113-1A in glucose-limited chemostat, D = 0.1 h^-1^, in different oxygenation conditions. The fragmentomers were obtained using biosynthetic fractional [U-^13^C]glucose labelling during metabolic steady state, ^13^C-HSQC NMR measurements and software FCAL for the integration of ^13^C-scalar fine structures of the amino acid carbon signals in the ^13^C-HSQC NMR spectra and the calculation of relative abundances of fragmentomers using probabilistic equations relating the ^13^C-scalar fine structures and the intact carbon chain fragments [43]. The *f*-values for the replicate experiments and their standard deviations are given in columns. For nomenclature of *f*-values, see Methods. Click here for file

Additional file 2 **Confidence intervals (95%) for the metabolic net fluxes**. 95% confidence intervals for the net fluxes in the central carbon of *S. cerevisiae *CEN.PK113-1A in glucose-limited chemostats, D = 0.1 h^-1^, in different oxygenation conditions. The lower and upper bounds for the confidence intervals of the replicate experiments are given in μmol/(g CDW h) for each net flux. ND stands for not determined because the reactions were excluded from the stoichiometric models according to the metabolic flux ratio (METAFoR) analysis data. Click here for file

Additional file 3 **Pearson correlations between the transcriptional levels of genes encoding metabolic enzymes and the corresponding fluxes**. The Pearson correlation between the transcriptional levels of the *S. cerevisiae *genes encoding metabolic enzymes, whose levels were measured in Wiebe *et al. *[32], mapped to the corresponding fluxes in the ^13^C-MFA model, and flux values in five different oxygenation conditions (0, 0.5, 1.0, 2.8 and 20.9% oxygen). The pairs of genes and fluxes that showed positively correlated behaviour (Pearson correlation >0.60) are marked with green. Click here for file

Additional file 4 **Stoichiometric model of the central carbon metabolism of *S. cerevisiae***. Reactions in the stoichiometric model of the central carbon metabolism of *Saccharomyces cerevisiae*, including also anabolic fluxes from metabolic intermediates to biosynthesis, transport reactions across the mitochondrial membrane and uptake and excretion reactions, applied in the ^13^C-MFA determination of the metabolic net fluxes in different oxygenation conditions. Click here for file
