# Supplementary material for: Oxygen dependence of metabolic fluxes and energy generation of Saccharomyces cerevisiae CEN.PK113-1A
Source: BMC Syst Biol. 2008 Jul 9;2:60. doi: 10.1186/1752-0509-2-60 (PMC2507709; doi:10.1186/1752-0509-2-60)
Supplement: Additional file 1 — Relative abundances of intact carbon fragments in proteinogenic amino acids. Relative abundances of intact C2 and C3 fragments (f-values) in proteinogenic amino acids describing the conservation of carbon chain fragments in the metabolism of S. cerevisiae CEN.PK113-1A in glucose-limited chemostat, D = 0.1 h-1, in different oxygenation conditions. The fragmentomers were obtained using biosynthetic fractional [U-13C]glucose labelling during metabolic steady state, 13C-HSQC NMR measurements and software FCAL for the integration of 13C-scalar fine structures of the amino acid carbon signals in the 13C-HSQC NMR spectra and the calculation of relative abundances of fragmentomers using probabilistic equations relating the 13C-scalar fine structures and the intact carbon chain fragments [43]. The f-values for the replicate experiments and their standard deviations are given in columns. For nomenclature of f-values, see Methods. [file 1752-0509-2-60-S1.doc]

|  |  |  | O_2_ provided in fermentor inlet gas | | | | | | | | | | | | | | | | | | | | | | | |
| --- | --- | --- | --- | --- | --- | --- | --- | --- | --- | --- | --- | --- | --- | --- | --- | --- | --- | --- | --- | --- | --- | --- | --- | --- | --- | --- |
|  |  |  | 20.9% | | | |  | 2.8% | | | |  | 1.0% | | | |  | 0.5% | | | |  | 0.0% | | | |
|  |  |  | I | stdv | II | stdv |  | I | stdv | II | stdv |  | I | stdv | II | stdv |  | I | stdv | II | stdv |  | I | stdv | II | stdv |
| ALA | CA: | f^(1)^ | 0.05 | 0.02 | 0.01 | 0.02 |  | 0.03 | 0.00 | 0.04 | 0.00 |  | 0.02 | 0.01 | 0.00 | 0.00 |  | 0.01 | 0.01 | 0.02 | 0.00 |  | 0.02 | 0.00 | 0.00 | 0.00 |
|  |  | f^(2)^ | 0.05 | 0.02 | 0.15 | 0.01 |  | 0.06 | 0.01 | 0.09 | 0.01 |  | 0.04 | 0.01 | 0.04 | 0.01 |  | 0.06 | 0.00 | 0.03 | 0.01 |  | 0.06 | 0.00 | 0.06 | 0.01 |
|  |  | f^(2*)^ | 0.02 | 0.01 | 0.00 | 0.00 |  | 0.01 | 0.01 | 0.00 | 0.00 |  | 0.00 | 0.00 | 0.00 | 0.00 |  | 0.00 | 0.00 | 0.00 | 0.00 |  | 0.00 | 0.00 | 0.00 | 0.00 |
|  |  | f^(3)^ | 0.88 | 0.03 | 0.84 | 0.02 |  | 0.90 | 0.01 | 0.87 | 0.01 |  | 0.94 | 0.01 | 0.96 | 0.01 |  | 0.93 | 0.01 | 0.95 | 0.01 |  | 0.92 | 0.01 | 0.94 | 0.01 |
|  |  |  |  |  |  |  |  |  |  |  |  |  |  |  |  |  |  |  |  |  |  |  |  |  |  |  |
| ALA | CB: | f^(1)^ | 0.08 | 0.00 | 0.07 | 0.00 |  | 0.07 | 0.00 | 0.06 | 0.00 |  | 0.05 | 0.00 | 0.02 | 0.00 |  | 0.04 | 0.00 | 0.03 | 0.00 |  | 0.02 | 0.00 | 0.02 | 0.00 |
|  |  | f^(2)^ | 0.92 | 0.00 | 0.93 | 0.00 |  | 0.93 | 0.00 | 0.94 | 0.00 |  | 0.95 | 0.00 | 0.98 | 0.00 |  | 0.96 | 0.00 | 0.97 | 0.00 |  | 0.98 | 0.00 | 0.98 | 0.00 |
|  |  |  |  |  |  |  |  |  |  |  |  |  |  |  |  |  |  |  |  |  |  |  |  |  |  |  |
| ARG | CB: | f^(1)^ | 0.71 | 0.03 | 0.64 | 0.03 |  | 0.68 | 0.03 | 0.71 | 0.02 |  | 0.64 | 0.02 | 0.62 | 0.01 |  | 0.53 | 0.02 | 0.45 | 0.01 |  | 0.03 | 0.01 | 0.04 | 0.01 |
|  |  | f^(2)^ | 0.28 | 0.01 | 0.32 | 0.01 |  | 0.31 | 0.02 | 0.29 | 0.01 |  | 0.35 | 0.01 | 0.37 | 0.02 |  | 0.47 | 0.07 | 0.55 | 0.16 |  | 0.97 | 0.04 | 0.96 | 0.02 |
|  |  | f^(2*)^ | 0.00 | 0.00 | 0.00 | 0.00 |  | 0.00 | 0.00 | 0.00 | 0.00 |  | 0.00 | 0.00 | 0.00 | 0.00 |  | 0.00 | 0.00 | 0.00 | 0.00 |  | 0.00 | 0.00 | 0.00 | 0.00 |
|  |  | f^(3)^ | 0.01 | 0.03 | 0.04 | 0.03 |  | 0.01 | 0.02 | 0.00 | 0.01 |  | 0.00 | 0.01 | 0.00 | 0.00 |  | 0.00 | 0.00 | 0.00 | 0.01 |  | 0.00 | 0.00 | 0.00 | 0.00 |
|  |  |  |  |  |  |  |  |  |  |  |  |  |  |  |  |  |  |  |  |  |  |  |  |  |  |  |
| ARG | CD: | f^(1)^ | 0.15 | 0.02 | 0.11 | 0.01 |  | 0.11 | 0.00 | 0.67 | 0.00 |  | 0.10 | 0.00 | 0.09 | 0.00 |  | 0.09 | 0.01 | 0.14 | 0.00 |  | 0.08 | 0.00 | 0.07 | 0.00 |
|  |  | f^(2)^ | 0.85 | 0.02 | 0.89 | 0.01 |  | 0.89 | 0.00 | 0.33 | 0.00 |  | 0.90 | 0.00 | 0.91 | 0.00 |  | 0.91 | 0.01 | 0.86 | 0.00 |  | 0.92 | 0.00 | 0.93 | 0.00 |
|  |  |  |  |  |  |  |  |  |  |  |  |  |  |  |  |  |  |  |  |  |  |  |  |  |  |  |
| ASP | CA: | f^(1)^ | 0.21 | 0.00 | 0.20 | 0.01 |  | 0.19 | 0.00 | 0.22 | 0.00 |  | 0.13 | 0.00 | 0.12 | 0.00 |  | 0.08 | 0.00 | 0.08 | 0.00 |  | 0.02 | 0.00 | 0.01 | 0.00 |
|  |  | f^(2)^ | 0.09 | 0.00 | 0.10 | 0.00 |  | 0.09 | 0.00 | 0.09 | 0.00 |  | 0.07 | 0.00 | 0.07 | 0.00 |  | 0.08 | 0.00 | 0.10 | 0.00 |  | 0.13 | 0.00 | 0.14 | 0.01 |
|  |  | f^(2*)^ | 0.23 | 0.00 | 0.25 | 0.00 |  | 0.26 | 0.00 | 0.26 | 0.00 |  | 0.15 | 0.00 | 0.17 | 0.00 |  | 0.14 | 0.00 | 0.13 | 0.00 |  | 0.00 | 0.00 | 0.00 | 0.00 |
|  |  | f^(3)^ | 0.46 | 0.00 | 0.45 | 0.01 |  | 0.47 | 0.00 | 0.43 | 0.00 |  | 0.65 | 0.00 | 0.64 | 0.00 |  | 0.69 | 0.00 | 0.69 | 0.01 |  | 0.85 | 0.00 | 0.85 | 0.00 |
|  |  |  |  |  |  |  |  |  |  |  |  |  |  |  |  |  |  |  |  |  |  |  |  |  |  |  |
| ASP | CB: | f^(1)^ | 0.21 | 0.01 | 0.21 | 0.00 |  | 0.19 | 0.00 | 0.22 | 0.00 |  | 0.13 | 0.00 | 0.12 | 0.00 |  | 0.09 | 0.00 | 0.09 | 0.00 |  | 0.01 | 0.00 | 0.01 | 0.00 |
|  |  | f^(2)^ | 0.54 | 0.01 | 0.48 | 0.00 |  | 0.52 | 0.00 | 0.47 | 0.00 |  | 0.69 | 0.00 | 0.66 | 0.00 |  | 0.73 | 0.00 | 0.74 | 0.00 |  | 0.89 | 0.00 | 0.89 | 0.00 |
|  |  | f^(2*)^ | 0.22 | 0.01 | 0.25 | 0.00 |  | 0.25 | 0.00 | 0.24 | 0.00 |  | 0.16 | 0.00 | 0.17 | 0.00 |  | 0.15 | 0.00 | 0.12 | 0.00 |  | 0.00 | 0.00 | 0.00 | 0.00 |
|  |  | f^(3)^ | 0.04 | 0.01 | 0.07 | 0.00 |  | 0.05 | 0.00 | 0.07 | 0.00 |  | 0.02 | 0.00 | 0.06 | 0.00 |  | 0.04 | 0.00 | 0.05 | 0.00 |  | 0.09 | 0.00 | 0.10 | 0.00 |
|  |  |  |  |  |  |  |  |  |  |  |  |  |  |  |  |  |  |  |  |  |  |  |  |  |  |  |
| GLU | CA: | f^(1)^ | 0.30 | 0.00 | 0.27 | 0.01 |  | 0.29 | 0.00 | 0.30 | 0.00 |  | 0.27 | 0.00 | 0.24 | 0.00 |  | 0.18 | 0.01 | 0.13 | 0.00 |  | 0.02 | 0.00 | 0.02 | 0.00 |
|  |  | f^(2)^ | 0.19 | 0.00 | 0.19 | 0.01 |  | 0.18 | 0.01 | 0.20 | 0.00 |  | 0.23 | 0.00 | 0.26 | 0.00 |  | 0.31 | 0.00 | 0.36 | 0.00 |  | 0.83 | 0.00 | 0.84 | 0.00 |
|  |  | f^(2*)^ | 0.43 | 0.00 | 0.43 | 0.01 |  | 0.42 | 0.00 | 0.41 | 0.00 |  | 0.40 | 0.00 | 0.40 | 0.00 |  | 0.37 | 0.00 | 0.33 | 0.00 |  | 0.01 | 0.00 | 0.01 | 0.00 |
|  |  | f^(3)^ | 0.09 | 0.00 | 0.12 | 0.01 |  | 0.10 | 0.01 | 0.09 | 0.00 |  | 0.10 | 0.00 | 0.11 | 0.00 |  | 0.14 | 0.01 | 0.19 | 0.00 |  | 0.14 | 0.00 | 0.13 | 0.00 |
|  |  |  |  |  |  |  |  |  |  |  |  |  |  |  |  |  |  |  |  |  |  |  |  |  |  |  |
| GLU | CB: | f^(1)^ | 0.70 | 0.00 | 0.67 | 0.01 |  | 0.70 | 0.01 | 0.67 | 0.00 |  | 0.65 | 0.00 | 0.61 | 0.00 |  | 0.55 | 0.00 | 0.43 | 0.00 |  | 0.01 | 0.00 | 0.02 | 0.00 |
|  |  | f^(2)^ | 0.31 | 0.00 | 0.33 | 0.00 |  | 0.31 | 0.01 | 0.33 | 0.00 |  | 0.35 | 0.00 | 0.40 | 0.00 |  | 0.45 | 0.01 | 0.56 | 0.01 |  | 0.99 | 0.01 | 0.98 | 0.02 |
|  |  | f^(2*)^ | 0.00 | 0.00 | 0.00 | 0.00 |  | 0.00 | 0.00 | 0.00 | 0.00 |  | 0.00 | 0.00 | 0.00 | 0.00 |  | 0.00 | 0.00 | 0.00 | 0.00 |  | 0.00 | 0.00 | 0.00 | 0.00 |
|  |  | f^(3)^ | 0.00 | 0.00 | 0.00 | 0.00 |  | 0.00 | 0.00 | 0.00 | 0.00 |  | 0.00 | 0.00 | 0.00 | 0.00 |  | 0.00 | 0.00 | 0.02 | 0.00 |  | 0.00 | 0.00 | 0.00 | 0.00 |
|  |  |  |  |  |  |  |  |  |  |  |  |  |  |  |  |  |  |  |  |  |  |  |  |  |  |  |
| GLU | CG: | f^(1)^ | 0.08 | 0.00 | 0.02 | 0.00 |  | 0.06 | 0.00 | 0.08 | 0.00 |  | 0.06 | 0.00 | 0.03 | 0.00 |  | 0.03 | 0.00 | 0.06 | 0.00 |  | 0.04 | 0.00 | 0.04 | 0.00 |
|  |  | f^(2)^ | 0.01 | 0.00 | 0.00 | 0.00 |  | 0.00 | 0.00 | 0.00 | 0.00 |  | 0.00 | 0.00 | 0.00 | 0.00 |  | 0.01 | 0.00 | 0.00 | 0.00 |  | 0.00 | 0.00 | 0.00 | 0.00 |
|  |  | f^(2*)^ | 0.91 | 0.00 | 0.96 | 0.01 |  | 0.94 | 0.00 | 0.92 | 0.00 |  | 0.95 | 0.00 | 0.97 | 0.00 |  | 0.97 | 0.00 | 0.94 | 0.00 |  | 0.96 | 0.00 | 0.96 | 0.00 |
|  |  | f^(3)^ | 0.00 | 0.00 | 0.02 | 0.00 |  | 0.00 | 0.00 | 0.00 | 0.00 |  | 0.00 | 0.00 | 0.00 | 0.00 |  | 0.00 | 0.00 | 0.00 | 0.00 |  | 0.00 | 0.00 | 0.00 | 0.00 |
|  |  |  |  |  |  |  |  |  |  |  |  |  |  |  |  |  |  |  |  |  |  |  |  |  |  |  |
| GLY | CA: | f^(1)^ | 0.16 | 0.00 | 0.12 | 0.01 |  | 0.11 | 0.00 | 0.13 | 0.00 |  | 0.11 | 0.00 | 0.07 | 0.00 |  | 0.05 | 0.02 | 0.09 | 0.00 |  | 0.09 | 0.00 | 0.08 | 0.00 |
|  |  | f^(2)^ | 0.84 | 0.00 | 0.88 | 0.01 |  | 0.89 | 0.00 | 0.87 | 0.00 |  | 0.90 | 0.00 | 0.93 | 0.00 |  | 0.95 | 0.02 | 0.91 | 0.00 |  | 0.91 | 0.00 | 0.93 | 0.00 |
|  |  |  |  |  |  |  |  |  |  |  |  |  |  |  |  |  |  |  |  |  |  |  |  |  |  |  |
| HIS | CA: | f^(1)^ | 0.06 | 0.01 | 0.02 | 0.01 |  | 0.03 | 0.02 | 0.02 | 0.01 |  | 0.04 | 0.01 | 0.01 | 0.00 |  | 0.03 | 0.01 | 0.05 | 0.02 |  | 0.02 | 0.01 | 0.02 | 0.01 |
|  |  | f^(2)^ | 0.00 | 0.01 | 0.00 | 0.00 |  | 0.00 | 0.02 | 0.00 | 0.00 |  | 0.00 | 0.00 | 0.00 | 0.00 |  | 0.01 | 0.02 | 0.00 | 0.01 |  | 0.00 | 0.01 | 0.00 | 0.00 |
|  |  | f^(2*)^ | 0.04 | 0.01 | 0.03 | 0.02 |  | 0.03 | 0.02 | 0.02 | 0.01 |  | 0.01 | 0.01 | 0.02 | 0.00 |  | 0.02 | 0.02 | 0.01 | 0.02 |  | 0.00 | 0.01 | 0.00 | 0.00 |
|  |  | f^(3)^ | 0.90 | 0.02 | 0.95 | 0.02 |  | 0.94 | 0.02 | 0.96 | 0.01 |  | 0.95 | 0.01 | 0.97 | 0.01 |  | 0.94 | 0.04 | 0.93 | 0.03 |  | 0.98 | 0.02 | 0.98 | 0.01 |
|  |  |  |  |  |  |  |  |  |  |  |  |  |  |  |  |  |  |  |  |  |  |  |  |  |  |  |
| HIS | CB: | f^(1)^ | 0.09 | 0.01 | 0.04 | 0.03 |  | 0.05 | 0.01 | 0.07 | 0.01 |  | 0.05 | 0.01 | 0.06 | 0.01 |  | 0.11 | 0.01 | 0.11 | 0.01 |  | 0.06 | 0.01 | 0.06 | 0.01 |
|  |  | f^(2)^ | 0.44 | 0.02 | 0.47 | 0.04 |  | 0.60 | 0.04 | 0.57 | 0.03 |  | 0.70 | 0.02 | 0.70 | 0.02 |  | 0.76 | 0.05 | 0.75 | 0.03 |  | 0.86 | 0.02 | 0.82 | 0.04 |
|  |  | f^(2*)^ | 0.01 | 0.04 | 0.03 | 0.04 |  | 0.00 | 0.02 | 0.02 | 0.02 |  | 0.00 | 0.00 | 0.02 | 0.01 |  | 0.00 | 0.01 | 0.00 | 0.01 |  | 0.00 | 0.00 | 0.01 | 0.02 |
|  |  | f^(3)^ | 0.46 | 0.03 | 0.46 | 0.05 |  | 0.35 | 0.03 | 0.34 | 0.02 |  | 0.25 | 0.02 | 0.22 | 0.01 |  | 0.13 | 0.06 | 0.14 | 0.03 |  | 0.08 | 0.02 | 0.11 | 0.05 |
|  |  |  |  |  |  |  |  |  |  |  |  |  |  |  |  |  |  |  |  |  |  |  |  |  |  |  |
| HIS | CD2: | f^(1)^ | 0.35 | 0.00 | 0.36 | 0.01 |  | 0.28 | 0.00 | 0.26 | 0.00 |  | 0.28 | 0.00 | 0.24 | 0.00 |  | 0.26 | 0.00 | 0.25 | 0.00 |  | 0.14 | 0.00 | 0.15 | 0.00 |
|  |  | f^(2)^ | 0.65 | 0.00 | 0.64 | 0.01 |  | 0.72 | 0.00 | 0.74 | 0.00 |  | 0.72 | 0.00 | 0.76 | 0.00 |  | 0.74 | 0.00 | 0.75 | 0.00 |  | 0.87 | 0.00 | 0.85 | 0.00 |
|  |  |  |  |  |  |  |  |  |  |  |  |  |  |  |  |  |  |  |  |  |  |  |  |  |  |  |
| ILE | CA: | f^(1)^ | 0.28 | 0.01 | 0.27 | 0.01 |  | 0.28 | 0.00 | 0.30 | 0.00 |  | 0.19 | 0.00 | 0.18 | 0.00 |  | 0.16 | 0.01 | 0.20 | 0.01 |  | 0.13 | 0.00 | 0.13 | 0.00 |
|  |  | f^(2)^ | 0.00 | 0.01 | 0.00 | 0.01 |  | 0.00 | 0.00 | 0.00 | 0.00 |  | 0.00 | 0.00 | 0.00 | 0.00 |  | 0.00 | 0.00 | 0.00 | 0.00 |  | 0.00 | 0.00 | 0.00 | 0.00 |
|  |  | f^(2*)^ | 0.72 | 0.01 | 0.65 | 0.01 |  | 0.72 | 0.00 | 0.71 | 0.00 |  | 0.81 | 0.00 | 0.82 | 0.00 |  | 0.84 | 0.01 | 0.81 | 0.01 |  | 0.87 | 0.00 | 0.88 | 0.00 |
|  |  | f^(3)^ | 0.00 | 0.02 | 0.07 | 0.01 |  | 0.00 | 0.00 | 0.00 | 0.00 |  | 0.00 | 0.00 | 0.00 | 0.00 |  | 0.00 | 0.00 | 0.00 | 0.00 |  | 0.00 | 0.00 | 0.00 | 0.00 |
|  |  |  |  |  |  |  |  |  |  |  |  |  |  |  |  |  |  |  |  |  |  |  |  |  |  |  |
| ILE | CG2: | f^(1)^ | 0.10 | 0.00 | 0.07 | 0.00 |  | 0.08 | 0.00 | 0.09 | 0.00 |  | 0.06 | 0.00 | 0.04 | 0.00 |  | 0.03 | 0.00 | 0.03 | 0.00 |  | 0.03 | 0.00 | 0.02 | 0.00 |
|  |  | f^(2)^ | 0.90 | 0.00 | 0.93 | 0.00 |  | 0.92 | 0.00 | 0.91 | 0.00 |  | 0.94 | 0.00 | 0.96 | 0.00 |  | 0.97 | 0.00 | 0.97 | 0.00 |  | 0.97 | 0.00 | 0.98 | 0.00 |
|  |  |  |  |  |  |  |  |  |  |  |  |  |  |  |  |  |  |  |  |  |  |  |  |  |  |  |
| ILE | CG1: | f^(1)^ | 0.76 | 0.03 | 0.65 | 0.03 |  | 0.69 | 0.02 | 0.71 | 0.01 |  | 0.86 | 0.01 | 0.81 | 0.01 |  | 0.80 | 0.02 | 0.84 | 0.01 |  | 0.91 | 0.02 | 0.87 | 0.02 |
|  |  | f^(2)^ | 0.24 | 0.03 | 0.29 | 0.03 |  | 0.31 | 0.21 | 0.29 | 0.01 |  | 0.13 | 0.02 | 0.19 | 0.01 |  | 0.21 | 0.02 | 0.15 | 0.01 |  | 0.08 | 0.01 | 0.12 | 0.01 |
|  |  | f^(2*)^ | 0.00 | 0.00 | 0.00 | 0.00 |  | 0.00 | 0.00 | 0.00 | 0.00 |  | 0.00 | 0.00 | 0.00 | 0.00 |  | 0.00 | 0.00 | 0.00 | 0.00 |  | 0.00 | 0.00 | 0.00 | 0.00 |
|  |  | f^(3)^ | 0.00 | 0.00 | 0.06 | 0.02 |  | 0.00 | 0.00 | 0.00 | 0.00 |  | 0.00 | 0.00 | 0.00 | 0.00 |  | 0.00 | 0.00 | 0.01 | 0.01 |  | 0.01 | 0.01 | 0.01 | 0.01 |
|  |  |  |  |  |  |  |  |  |  |  |  |  |  |  |  |  |  |  |  |  |  |  |  |  |  |  |
| ILE | CD1: | f^(1)^ | 0.77 | 0.00 | 0.86 | 0.01 |  | 0.70 | 0.00 | 0.69 | 0.00 |  | 0.85 | 0.00 | 0.84 | 0.00 |  | 0.96 | 0.01 | 0.83 | 0.01 |  | 0.96 | 0.01 | 1.00 | 0.00 |
|  |  | f^(2)^ | 0.23 | 0.00 | 0.14 | 0.01 |  | 0.30 | 0.00 | 0.31 | 0.00 |  | 0.15 | 0.00 | 0.16 | 0.00 |  | 0.04 | 0.01 | 0.17 | 0.01 |  | 0.04 | 0.01 | 0.00 | 0.00 |
|  |  |  |  |  |  |  |  |  |  |  |  |  |  |  |  |  |  |  |  |  |  |  |  |  |  |  |
| LEU | CA: | f^(1)^ | 0.07 | 0.00 | 0.05 | 0.01 |  | 0.06 | 0.00 | 0.08 | 0.01 |  | 0.07 | 0.00 | 0.04 | 0.01 |  | 0.05 | 0.00 | 0.10 | 0.01 |  | 0.05 | 0.00 | 0.05 | 0.00 |
|  |  | f^(2)^ | 0.03 | 0.01 | 0.03 | 0.01 |  | 0.00 | 0.00 | 0.00 | 0.00 |  | 0.00 | 0.00 | 0.00 | 0.00 |  | 0.00 | 0.00 | 0.00 | 0.00 |  | 0.00 | 0.00 | 0.00 | 0.00 |
|  |  | f^(2*)^ | 0.90 | 0.01 | 0.92 | 0.01 |  | 0.94 | 0.00 | 0.92 | 0.01 |  | 0.93 | 0.00 | 0.96 | 0.01 |  | 0.95 | 0.00 | 0.90 | 0.01 |  | 0.95 | 0.00 | 0.95 | 0.00 |
|  |  | f^(3)^ | 0.00 | 0.00 | 0.01 | 0.02 |  | 0.00 | 0.00 | 0.00 | 0.00 |  | 0.00 | 0.00 | 0.00 | 0.00 |  | 0.00 | 0.00 | 0.00 | 0.00 |  | 0.00 | 0.00 | 0.00 | 0.00 |
|  |  |  |  |  |  |  |  |  |  |  |  |  |  |  |  |  |  |  |  |  |  |  |  |  |  |  |
| LEU | CD1: | f^(1)^ | 0.14 | 0.00 | 0.10 | 0.00 |  | 0.13 | 0.00 | 0.15 | 0.00 |  | 0.11 | 0.00 | 0.06 | 0.00 |  | 0.03 | 0.00 | 0.07 | 0.00 |  | 0.05 | 0.00 | 0.03 | 0.00 |
|  |  | f^(2)^ | 0.86 | 0.00 | 0.90 | 0.00 |  | 0.87 | 0.00 | 0.85 | 0.00 |  | 0.90 | 0.00 | 0.94 | 0.00 |  | 0.97 | 0.00 | 0.93 | 0.00 |  | 0.95 | 0.00 | 0.97 | 0.00 |
|  |  |  |  |  |  |  |  |  |  |  |  |  |  |  |  |  |  |  |  |  |  |  |  |  |  |  |
| LEU | CD2: | f^(1)^ | 1.00 | 0.00 | 1.00 | 0.02 |  | 0.99 | 0.00 | 1.00 | 0.00 |  | 1.00 | 0.00 | 1.00 | 0.00 |  | 1.00 | 0.00 | 1.00 | 0.00 |  | 1.00 | 0.00 | 1.00 | 0.00 |
|  |  | f^(2)^ | 0.00 | 0.00 | 0.00 | 0.02 |  | 0.01 | 0.00 | 0.00 | 0.00 |  | 0.01 | 0.00 | 0.00 | 0.00 |  | 0.00 | 0.00 | 0.00 | 0.00 |  | 0.00 | 0.00 | 0.01 | 0.00 |
|  |  |  |  |  |  |  |  |  |  |  |  |  |  |  |  |  |  |  |  |  |  |  |  |  |  |  |
| LYS | CA: | f^(1)^ | 0.07 | 0.00 | 0.06 | 0.00 |  | 0.05 | 0.00 | 0.04 | 0.01 |  | 0.06 | 0.01 | 0.04 | 0.00 |  | 0.02 | 0.01 | 0.22 | 0.28 |  | 0.04 | 0.00 | 0.02 | 0.01 |
|  |  | f^(2)^ | 0.00 | 0.00 | 0.00 | 0.00 |  | 0.00 | 0.00 | 0.00 | 0.00 |  | 0.00 | 0.00 | 0.00 | 0.00 |  | 0.00 | 0.00 | 0.00 | 0.00 |  | 0.00 | 0.00 | 0.00 | 0.00 |
|  |  | f^(2*)^ | 0.94 | 0.00 | 0.94 | 0.04 |  | 0.95 | 0.00 | 0.96 | 0.01 |  | 0.94 | 0.01 | 0.94 | 0.01 |  | 0.98 | 0.01 | 0.71 | 0.40 |  | 0.96 | 0.01 | 0.98 | 0.02 |
|  |  | f^(3)^ | 0.00 | 0.00 | 0.00 | 0.01 |  | 0.00 | 0.00 | 0.00 | 0.00 |  | 0.00 | 0.00 | 0.02 | 0.00 |  | 0.00 | 0.00 | 0.08 | 0.22 |  | 0.00 | 0.00 | 0.00 | 0.01 |
|  |  |  |  |  |  |  |  |  |  |  |  |  |  |  |  |  |  |  |  |  |  |  |  |  |  |  |
| LYS | CE: | f^(1)^ | 0.16 | 0.00 | 0.12 | 0.00 |  | 0.12 | 0.00 | 0.15 | 0.00 |  | 0.10 | 0.00 | 0.11 | 0.00 |  | 0.09 | 0.00 | 0.14 | 0.00 |  | 0.08 | 0.00 | 0.07 | 0.00 |
|  |  | f^(2)^ | 0.84 | 0.00 | 0.88 | 0.00 |  | 0.88 | 0.00 | 0.85 | 0.00 |  | 0.90 | 0.00 | 0.89 | 0.00 |  | 0.91 | 0.00 | 0.86 | 0.00 |  | 0.92 | 0.00 | 0.93 | 0.00 |
|  |  |  |  |  |  |  |  |  |  |  |  |  |  |  |  |  |  |  |  |  |  |  |  |  |  |  |
| MET | CA: | f^(1)^ | 0.20 | 0.00 | 0.16 | 0.06 |  | 0.19 | 0.01 | 0.21 | 0.02 |  | 0.13 | 0.02 | 0.11 | 0.02 |  | 0.10 | 0.01 | 0.09 | 0.03 |  | 0.01 | 0.01 | 0.04 | 0.01 |
|  |  | f^(2)^ | 0.11 | 0.01 | 0.15 | 0.11 |  | 0.12 | 0.03 | 0.11 | 0.02 |  | 0.11 | 0.02 | 0.10 | 0.02 |  | 0.11 | 0.03 | 0.11 | 0.01 |  | 0.08 | 0.01 | 0.12 | 0.02 |
|  |  | f^(2*)^ | 0.22 | 0.01 | 0.22 | 0.05 |  | 0.25 | 0.01 | 0.25 | 0.01 |  | 0.15 | 0.01 | 0.15 | 0.02 |  | 0.13 | 0.02 | 0.11 | 0.02 |  | 0.00 | 0.01 | 0.00 | 0.01 |
|  |  | f^(3)^ | 0.48 | 0.01 | 0.47 | 0.07 |  | 0.45 | 0.03 | 0.43 | 0.02 |  | 0.61 | 0.01 | 0.64 | 0.05 |  | 0.66 | 0.04 | 0.70 | 0.03 |  | 0.90 | 0.01 | 0.84 | 0.02 |
|  |  |  |  |  |  |  |  |  |  |  |  |  |  |  |  |  |  |  |  |  |  |  |  |  |  |  |
| PHE | CA: | f^(1)^ | 0.05 | 0.01 | 0.03 | 0.01 |  | 0.02 | 0.00 | 0.02 | 0.00 |  | 0.03 | 0.00 | 0.01 | 0.00 |  | 0.03 | 0.01 | 0.04 | 0.01 |  | 0.02 | 0.00 | 0.02 | 0.00 |
|  |  | f^(2)^ | 0.07 | 0.02 | 0.08 | 0.02 |  | 0.06 | 0.01 | 0.05 | 0.00 |  | 0.04 | 0.01 | 0.05 | 0.01 |  | 0.03 | 0.01 | 0.01 | 0.01 |  | 0.01 | 0.00 | 0.02 | 0.01 |
|  |  | f^(2*)^ | 0.00 | 0.00 | 0.01 | 0.01 |  | 0.01 | 0.00 | 0.00 | 0.00 |  | 0.01 | 0.00 | 0.00 | 0.00 |  | 0.01 | 0.01 | 0.00 | 0.01 |  | 0.00 | 0.00 | 0.00 | 0.01 |
|  |  | f^(3)^ | 0.88 | 0.03 | 0.88 | 0.02 |  | 0.92 | 0.01 | 0.92 | 0.01 |  | 0.93 | 0.01 | 0.94 | 0.01 |  | 0.93 | 0.01 | 0.94 | 0.01 |  | 0.96 | 0.00 | 0.96 | 0.01 |
|  |  |  |  |  |  |  |  |  |  |  |  |  |  |  |  |  |  |  |  |  |  |  |  |  |  |  |
| PHE | CB: | f^(1)^ | 0.04 | 0.00 | 0.02 | 0.01 |  | 0.02 | 0.01 | 0.01 | 0.01 |  | 0.04 | 0.01 | 0.01 | 0.00 |  | 0.02 | 0.01 | 0.03 | 0.00 |  | 0.03 | 0.01 | 0.02 | 0.01 |
|  |  | f^(2)^ | 0.96 | 0.00 | 0.94 | 0.03 |  | 0.91 | 0.10 | 0.97 | 0.01 |  | 0.96 | 0.01 | 0.99 | 0.00 |  | 0.97 | 0.01 | 0.97 | 0.00 |  | 0.97 | 0.01 | 0.98 | 0.01 |
|  |  | f^(2*)^ | 0.00 | 0.00 | 0.01 | 0.02 |  | 0.07 | 0.10 | 0.02 | 0.01 |  | 0.00 | 0.01 | 0.00 | 0.00 |  | 0.00 | 0.00 | 0.00 | 0.00 |  | 0.00 | 0.00 | 0.00 | 0.00 |
|  |  | f^(3)^ | 0.00 | 0.00 | 0.04 | 0.03 |  | 0.00 | 0.00 | 0.00 | 0.00 |  | 0.00 | 0.00 | 0.00 | 0.00 |  | 0.00 | 0.00 | 0.00 | 0.00 |  | 0.00 | 0.00 | 0.00 | 0.01 |
|  |  |  |  |  |  |  |  |  |  |  |  |  |  |  |  |  |  |  |  |  |  |  |  |  |  |  |
| PRO | CA: | f^(1)^ | 0.31 | 0.01 | 0.29 | 0.03 |  | 0.29 | 0.02 | 0.30 | 0.01 |  | 0.28 | 0.02 | 0.24 | 0.01 |  | 0.18 | 0.02 | 0.18 | 0.01 |  | 0.03 | 0.01 | 0.02 | 0.01 |
|  |  | f^(2)^ | 0.19 | 0.01 | 0.20 | 0.02 |  | 0.19 | 0.01 | 0.20 | 0.01 |  | 0.26 | 0.02 | 0.27 | 0.01 |  | 0.32 | 0.03 | 0.37 | 0.02 |  | 0.87 | 0.02 | 0.86 | 0.03 |
|  |  | f^(2*)^ | 0.41 | 0.01 | 0.41 | 0.01 |  | 0.41 | 0.00 | 0.42 | 0.01 |  | 0.40 | 0.01 | 0.40 | 0.01 |  | 0.37 | 0.01 | 0.27 | 0.01 |  | 0.00 | 0.01 | 0.00 | 0.01 |
|  |  | f^(3)^ | 0.09 | 0.01 | 0.10 | 0.04 |  | 0.11 | 0.02 | 0.08 | 0.02 |  | 0.06 | 0.02 | 0.10 | 0.01 |  | 0.14 | 0.05 | 0.18 | 0.02 |  | 0.09 | 0.02 | 0.12 | 0.03 |
|  |  |  |  |  |  |  |  |  |  |  |  |  |  |  |  |  |  |  |  |  |  |  |  |  |  |  |
| PRO | CB: | f^(1)^ | 0.69 | 0.09 | 0.58 | 0.05 |  | 0.59 | 0.00 | 0.67 | 0.01 |  | 0.62 | 0.04 | 0.56 | 0.01 |  | 0.47 | 0.03 | 0.39 | 0.03 |  | 0.01 | 0.01 | 0.02 | 0.05 |
|  |  | f^(2)^ | 0.30 | 0.08 | 0.37 | 0.09 |  | 0.41 | 0.02 | 0.33 | 0.01 |  | 0.36 | 0.05 | 0.44 | 0.03 |  | 0.50 | 0.21 | 0.54 | 0.09 |  | 0.98 | 0.28 | 0.72 | 0.29 |
|  |  | f^(2*)^ | 0.00 | 0.00 | 0.00 | 0.00 |  | 0.00 | 0.00 | 0.00 | 0.00 |  | 0.00 | 0.00 | 0.00 | 0.00 |  | 0.00 | 0.00 | 0.00 | 0.00 |  | 0.00 | 0.00 | 0.00 | 0.00 |
|  |  | f^(3)^ | 0.01 | 0.01 | 0.05 | 0.08 |  | 0.00 | 0.00 | 0.01 | 0.01 |  | 0.02 | 0.03 | 0.00 | 0.00 |  | 0.03 | 0.03 | 0.04 | 0.02 |  | 0.02 | 0.02 | 0.03 | 0.04 |
|  |  |  |  |  |  |  |  |  |  |  |  |  |  |  |  |  |  |  |  |  |  |  |  |  |  |  |
| PRO | CG: | f^(1)^ | 0.09 | 0.02 | 0.07 | 0.02 |  | 0.02 | 0.02 | 0.08 | 0.00 |  | 0.06 | 0.01 | 0.05 | 0.00 |  | 0.05 | 0.01 | 0.13 | 0.02 |  | 0.04 | 0.00 | 0.05 | 0.02 |
|  |  | f^(2)^+f^(2*)^ | 0.78 | 0.25 | 0.91 | 0.04 |  | 0.82 | 0.05 | 0.91 | 0.03 |  | 0.87 | 0.04 | 0.93 | 0.02 |  | 0.91 | 0.05 | 0.80 | 0.15 |  | 0.90 | 0.04 | 0.87 | 0.05 |
|  |  | f^(3)^ | 0.03 | 0.04 | 0.02 | 0.03 |  | 0.15 | 0.02 | 0.01 | 0.01 |  | 0.06 | 0.02 | 0.02 | 0.01 |  | 0.04 | 0.02 | 0.01 | 0.02 |  | 0.06 | 0.01 | 0.05 | 0.02 |
|  |  |  |  |  |  |  |  |  |  |  |  |  |  |  |  |  |  |  |  |  |  |  |  |  |  |  |
| SER | CA: | f^(1)^ | 0.09 | 0.01 | 0.07 | 0.01 |  | 0.04 | 0.01 | 0.06 | 0.00 |  | 0.06 | 0.00 | 0.03 | 0.00 |  | 0.03 | 0.00 | 0.04 | 0.00 |  | 0.04 | 0.00 | 0.05 | 0.00 |
|  |  | f^(2)^ | 0.04 | 0.01 | 0.02 | 0.01 |  | 0.03 | 0.01 | 0.01 | 0.01 |  | 0.01 | 0.01 | 0.00 | 0.00 |  | 0.02 | 0.01 | 0.02 | 0.00 |  | 0.01 | 0.00 | 0.02 | 0.01 |
|  |  | f^(2*)^ | 0.32 | 0.01 | 0.34 | 0.01 |  | 0.34 | 0.00 | 0.34 | 0.00 |  | 0.33 | 0.00 | 0.36 | 0.00 |  | 0.36 | 0.00 | 0.39 | 0.01 |  | 0.39 | 0.00 | 0.39 | 0.00 |
|  |  | f^(3)^ | 0.55 | 0.03 | 0.57 | 0.01 |  | 0.59 | 0.01 | 0.58 | 0.00 |  | 0.60 | 0.00 | 0.61 | 0.00 |  | 0.58 | 0.00 | 0.56 | 0.01 |  | 0.55 | 0.00 | 0.54 | 0.00 |
|  |  |  |  |  |  |  |  |  |  |  |  |  |  |  |  |  |  |  |  |  |  |  |  |  |  |  |
| SER | CB: | f^(1)^ | 0.43 | 0.00 | 0.46 | 0.01 |  | 0.39 | 0.00 | 0.44 | 0.00 |  | 0.39 | 0.00 | 0.41 | 0.00 |  | 0.39 | 0.00 | 0.44 | 0.00 |  | 0.45 | 0.00 | 0.44 | 0.09 |
|  |  | f^(2)^ | 0.57 | 0.00 | 0.54 | 0.01 |  | 0.61 | 0.00 | 0.56 | 0.00 |  | 0.61 | 0.00 | 0.59 | 0.00 |  | 0.61 | 0.00 | 0.57 | 0.00 |  | 0.55 | 0.00 | 0.56 | 0.09 |
|  |  |  |  |  |  |  |  |  |  |  |  |  |  |  |  |  |  |  |  |  |  |  |  |  |  |  |
| THR | CA: | f^(1)^ | 0.21 | 0.01 | 0.19 | 0.01 |  | 0.20 | 0.01 | 0.21 | 0.00 |  | 0.14 | 0.01 | 0.12 | 0.00 |  | 0.10 | 0.00 | 0.10 | 0.00 |  | 0.03 | 0.00 | 0.02 | 0.01 |
|  |  | f^(2)^ | 0.09 | 0.00 | 0.09 | 0.01 |  | 0.09 | 0.01 | 0.09 | 0.00 |  | 0.06 | 0.00 | 0.07 | 0.00 |  | 0.08 | 0.00 | 0.10 | 0.00 |  | 0.12 | 0.00 | 0.13 | 0.00 |
|  |  | f^(2*)^ | 0.22 | 0.00 | 0.26 | 0.01 |  | 0.25 | 0.00 | 0.26 | 0.00 |  | 0.15 | 0.00 | 0.17 | 0.00 |  | 0.13 | 0.01 | 0.11 | 0.01 |  | 0.00 | 0.00 | 0.00 | 0.00 |
|  |  | f^(3)^ | 0.48 | 0.00 | 0.46 | 0.00 |  | 0.46 | 0.00 | 0.44 | 0.00 |  | 0.65 | 0.00 | 0.65 | 0.00 |  | 0.70 | 0.01 | 0.69 | 0.00 |  | 0.86 | 0.00 | 0.85 | 0.00 |
|  |  |  |  |  |  |  |  |  |  |  |  |  |  |  |  |  |  |  |  |  |  |  |  |  |  |  |
| THR | CB: | f^(1)^ | 0.22 | 0.00 | 0.22 | 0.01 |  | 0.20 | 0.01 | 0.23 | 0.00 |  | 0.11 | 0.00 | 0.11 | 0.00 |  | 0.09 | 0.01 | 0.08 | 0.01 |  | 0.05 | 0.00 | 0.00 | 0.00 |
|  |  | f^(2)^+f^(2*)^ | 0.76 | 0.01 | 0.76 | 0.03 |  | 0.76 | 0.00 | 0.75 | 0.00 |  | 0.87 | 0.04 | 0.88 | 0.00 |  | 0.88 | 0.12 | 0.86 | 0.06 |  | 0.91 | 0.01 | 0.90 | 0.01 |
|  |  | f^(3)^ | 0.02 | 0.01 | 0.02 | 0.02 |  | 0.04 | 0.01 | 0.03 | 0.01 |  | 0.02 | 0.01 | 0.01 | 0.00 |  | 0.03 | 0.01 | 0.06 | 0.01 |  | 0.05 | 0.01 | 0.10 | 0.01 |
|  |  |  |  |  |  |  |  |  |  |  |  |  |  |  |  |  |  |  |  |  |  |  |  |  |  |  |
| THR | CG2: | f^(1)^ | 0.78 | 0.00 | 0.73 | 0.00 |  | 0.70 | 0.00 | 0.68 | 0.00 |  | 0.84 | 0.00 | 0.81 | 0.00 |  | 0.82 | 0.00 | 0.83 | 0.00 |  | 0.95 | 0.00 | 0.94 | 0.00 |
|  |  | f^(2)^ | 0.22 | 0.00 | 0.27 | 0.00 |  | 0.30 | 0.00 | 0.32 | 0.00 |  | 0.16 | 0.00 | 0.19 | 0.00 |  | 0.18 | 0.00 | 0.17 | 0.00 |  | 0.05 | 0.00 | 0.06 | 0.00 |
|  |  |  |  |  |  |  |  |  |  |  |  |  |  |  |  |  |  |  |  |  |  |  |  |  |  |  |
| TYR | CA: | f^(1)^ | 0.05 | 0.01 | 0.03 | 0.01 |  | 0.02 | 0.01 | 0.02 | 0.01 |  | 0.04 | 0.01 | 0.02 | 0.00 |  | 0.04 | 0.01 | 0.05 | 0.01 |  | 0.04 | 0.00 | 0.02 | 0.01 |
|  |  | f^(2)^ | 0.07 | 0.02 | 0.07 | 0.03 |  | 0.05 | 0.01 | 0.05 | 0.02 |  | 0.04 | 0.01 | 0.05 | 0.01 |  | 0.03 | 0.01 | 0.02 | 0.01 |  | 0.02 | 0.01 | 0.02 | 0.01 |
|  |  | f^(2*)^ | 0.01 | 0.01 | 0.03 | 0.02 |  | 0.00 | 0.00 | 0.01 | 0.01 |  | 0.00 | 0.00 | 0.02 | 0.01 |  | 0.01 | 0.01 | 0.01 | 0.02 |  | 0.00 | 0.01 | 0.01 | 0.01 |
|  |  | f^(3)^ | 0.87 | 0.03 | 0.87 | 0.04 |  | 0.92 | 0.02 | 0.92 | 0.03 |  | 0.92 | 0.01 | 0.90 | 0.01 |  | 0.92 | 0.02 | 0.91 | 0.03 |  | 0.94 | 0.01 | 0.95 | 0.02 |
|  |  |  |  |  |  |  |  |  |  |  |  |  |  |  |  |  |  |  |  |  |  |  |  |  |  |  |
| TYR | CB: | f^(1)^ | 0.06 | 0.07 | 0.02 | 0.01 |  | 0.07 | 0.18 | 0.01 | 0.01 |  | 0.05 | 0.01 | 0.00 | 0.01 |  | 0.04 | 0.01 | 0.04 | 0.01 |  | 0.04 | 0.01 | 0.03 | 0.01 |
|  |  | f^(2)^ | 0.92 | 0.15 | 0.85 | 0.04 |  | 0.92 | 0.20 | 0.99 | 0.01 |  | 0.95 | 0.01 | 0.99 | 0.01 |  | 0.96 | 0.01 | 0.96 | 0.01 |  | 0.96 | 0.01 | 0.97 | 0.01 |
|  |  | f^(2*)^ | 0.00 | 0.00 | 0.04 | 0.02 |  | 0.00 | 0.01 | 0.00 | 0.00 |  | 0.00 | 0.00 | 0.00 | 0.01 |  | 0.00 | 0.00 | 0.00 | 0.01 |  | 0.00 | 0.00 | 0.00 | 0.00 |
|  |  | f^(3)^ | 0.03 | 0.09 | 0.09 | 0.03 |  | 0.01 | 0.02 | 0.00 | 0.00 |  | 0.00 | 0.00 | 0.00 | 0.00 |  | 0.00 | 0.00 | 0.00 | 0.00 |  | 0.00 | 0.00 | 0.00 | 0.00 |
|  |  |  |  |  |  |  |  |  |  |  |  |  |  |  |  |  |  |  |  |  |  |  |  |  |  |  |
| TYR | CD1: | f^(1)^ | 0.04 | 0.01 | 0.04 | 0.00 |  | 0.02 | 0.00 | 0.02 | 0.00 |  | 0.02 | 0.02 | 0.03 | 0.00 |  | 0.01 | 0.00 | 0.04 | 0.00 |  | 0.02 | 0.00 | 0.03 | 0.00 |
|  |  | f^(2)^+f^(2*)^ | 0.96 | 0.14 | 0.96 | 0.00 |  | 0.98 | 0.00 | 0.98 | 0.00 |  | 0.98 | 0.02 | 0.97 | 0.41 |  | 0.99 | 0.00 | 0.96 | 0.00 |  | 0.98 | 0.35 | 0.97 | 0.00 |
|  |  | f^(3)^ | 0.00 | 0.01 | 0.00 | 0.00 |  | 0.00 | 0.00 | 0.00 | 0.02 |  | 0.00 | 0.02 | 0.00 | 0.00 |  | 0.00 | 0.00 | 0.00 | 0.00 |  | 0.00 | 0.00 | 0.00 | 0.00 |
|  |  |  |  |  |  |  |  |  |  |  |  |  |  |  |  |  |  |  |  |  |  |  |  |  |  |  |
| TYR | CE1: | f^(1)^ | 0.30 | 0.01 | 0.27 | 0.00 |  | 0.27 | 0.00 | 0.30 | 0.00 |  | 0.26 | 0.02 | 0.29 | 0.01 |  | 0.26 | 0.01 | 0.29 | 0.00 |  | 0.28 | 0.00 | 0.29 | 0.00 |
|  |  | f^(2)^+f^(2*)^ | 0.25 | 0.02 | 0.25 | 0.00 |  | 0.24 | 0.00 | 0.22 | 0.00 |  | 0.26 | 0.04 | 0.28 | 0.01 |  | 0.25 | 0.02 | 0.24 | 0.00 |  | 0.23 | 0.00 | 0.25 | 0.00 |
|  |  | f^(3)^ | 0.46 | 0.01 | 0.48 | 0.00 |  | 0.50 | 0.00 | 0.48 | 0.00 |  | 0.48 | 0.02 | 0.43 | 0.01 |  | 0.49 | 0.01 | 0.47 | 0.00 |  | 0.49 | 0.00 | 0.47 | 0.00 |
|  |  |  |  |  |  |  |  |  |  |  |  |  |  |  |  |  |  |  |  |  |  |  |  |  |  |  |
| VAL | CA: | f^(1)^ | 0.14 | 0.01 | 0.12 | 0.01 |  | 0.10 | 0.00 | 0.11 | 0.00 |  | 0.07 | 0.00 | 0.05 | 0.00 |  | 0.04 | 0.01 | 0.07 | 0.00 |  | 0.10 | 0.00 | 0.09 | 0.00 |
|  |  | f^(2)^ | 0.00 | 0.00 | 0.00 | 0.01 |  | 0.00 | 0.00 | 0.00 | 0.00 |  | 0.00 | 0.00 | 0.00 | 0.00 |  | 0.00 | 0.00 | 0.00 | 0.01 |  | 0.00 | 0.00 | 0.00 | 0.00 |
|  |  | f^(2*)^ | 0.86 | 0.01 | 0.85 | 0.02 |  | 0.90 | 0.01 | 0.89 | 0.00 |  | 0.93 | 0.01 | 0.95 | 0.00 |  | 0.95 | 0.01 | 0.93 | 0.01 |  | 0.90 | 0.00 | 0.91 | 0.00 |
|  |  | f^(3)^ | 0.00 | 0.00 | 0.03 | 0.02 |  | 0.00 | 0.00 | 0.00 | 0.00 |  | 0.00 | 0.00 | 0.00 | 0.00 |  | 0.00 | 0.00 | 0.00 | 0.00 |  | 0.00 | 0.00 | 0.00 | 0.00 |
|  |  |  |  |  |  |  |  |  |  |  |  |  |  |  |  |  |  |  |  |  |  |  |  |  |  |  |
| VAL | CG1: | f^(1)^ | 0.11 | 0.00 | 0.06 | 0.00 |  | 0.08 | 0.00 | 0.06 | 0.00 |  | 0.09 | 0.00 | 0.04 | 0.00 |  | 0.05 | 0.00 | 0.04 | 0.00 |  | 0.02 | 0.00 | 0.01 | 0.00 |
|  |  | f^(2)^ | 0.89 | 0.00 | 0.94 | 0.00 |  | 0.92 | 0.00 | 0.94 | 0.00 |  | 0.91 | 0.00 | 0.96 | 0.00 |  | 0.95 | 0.00 | 0.96 | 0.00 |  | 0.98 | 0.00 | 1.00 | 0.00 |
|  |  |  |  |  |  |  |  |  |  |  |  |  |  |  |  |  |  |  |  |  |  |  |  |  |  |  |
| VAL | CG2: | f^(1)^ | 1.00 | 0.00 | 1.00 | 0.00 |  | 1.00 | 0.00 | 0.99 | 0.00 |  | 1.00 | 0.00 | 0.99 | 0.00 |  | 0.98 | 0.00 | 1.00 | 0.00 |  | 1.00 | 0.00 | 1.00 | 0.00 |
|  |  | f^(2)^ | 0.00 | 0.00 | 0.00 | 0.00 |  | 0.00 | 0.00 | 0.01 | 0.00 |  | 0.00 | 0.00 | 0.01 | 0.00 |  | 0.02 | 0.00 | 0.00 | 0.00 |  | 0.00 | 0.00 | 0.00 | 0.00 |
