# Supplementary material for: Oxygen dependence of metabolic fluxes and energy generation of Saccharomyces cerevisiae CEN.PK113-1A
Source: BMC Syst Biol. 2008 Jul 9;2:60. doi: 10.1186/1752-0509-2-60 (PMC2507709; doi:10.1186/1752-0509-2-60)
Supplement: Additional file 2 — Confidence intervals (95%) for the metabolic net fluxes. 95% confidence intervals for the net fluxes in the central carbon of S. cerevisiae CEN.PK113-1A in glucose-limited chemostats, D = 0.1 h-1, in different oxygenation conditions. The lower and upper bounds for the confidence intervals of the replicate experiments are given in μmol/(g CDW h) for each net flux. ND stands for not determined because the reactions were excluded from the stoichiometric models according to the metabolic flux ratio (METAFoR) analysis data. [file 1752-0509-2-60-S2.doc]

|  | O_2_ in the fermentor inlet gas | | | | | | | | | | | | | | | | | | | | | | | |
| --- | --- | --- | --- | --- | --- | --- | --- | --- | --- | --- | --- | --- | --- | --- | --- | --- | --- | --- | --- | --- | --- | --- | --- | --- |
| Flux | 20.9% | | | |  | 2.8% | | | |  | 1.0% | | | |  | 0.5% | | | |  | 0.0% | | | |
|  | I | | II | |  | I | | II | |  | I | | II | |  | I | | II | |  | I | | II | |
| x_i_ | low | high | low | high |  | low | high | low | high |  | low | high | low | high |  | low | high | low | high |  | low | high | low | high |
| 1 | 1264 | 1285 | 860 | 876 |  | 1346 | 1369 | 1267 | 1280 |  | 1955 | 1972 | 2089 | 2144 |  | 2145 | 2229 | 2604 | 2752 |  | 6173 | 6208 | 6489 | 6520 |
| 2 | 578 | 693 | 373 | 438 |  | 722 | 864 | 666 | 790 |  | 1225 | 1432 | 1315 | 1556 |  | 1543 | 1823 | 1856 | 2245 |  | 5410 | 5474 | 5688 | 5764 |
| 3 | 312 | 345 | 231 | 261 |  | 235 | 254 | 229 | 245 |  | 288 | 305 | 359 | 401 |  | 219 | 245 | 191 | 222 |  | 299 | 345 | 301 | 358 |
| 4 | 743 | 891 | 497 | 589 |  | 834 | 1001 | 776 | 922 |  | 1369 | 1603 | 1509 | 1796 |  | 1655 | 1955 | 1938 | 2343 |  | 5585 | 5623 | 5868 | 5911 |
| 5 | 99 | 115 | 74 | 87 |  | 72 | 84 | 72 | 82 |  | 89 | 101 | 113 | 135 |  | 69 | 81 | 54 | 67 |  | 96 | 112 | 96 | 116 |
| 6 | 66 | 83 | 50 | 64 |  | 40 | 53 | 38 | 50 |  | 55 | 70 | 81 | 106 |  | 41 | 55 | 23 | 37 |  | 50 | 68 | 48 | 70 |
| 7 | 99 | 115 | 74 | 87 |  | 72 | 84 | 71 | 82 |  | 88 | 102 | 112 | 136 |  | 68 | 81 | 53 | 67 |  | 96 | 111 | 96 | 115 |
| 8 | 1469 | 1782 | 984 | 1181 |  | 1629 | 1975 | 1508 | 1811 |  | 2712 | 3195 | 3024 | 3623 |  | 3266 | 3906 | 3789 | 4640 |  | 10082 | 10150 | 10574 | 10656 |
| 9 | 1464 | 1804 | 1004 | 1230 |  | 1599 | 1965 | 1447 | 1759 |  | 2666 | 3207 | 3177 | 3856 |  | 3372 | 4113 | 3646 | 4630 |  | 10000 | 10068 | 10488 | 10572 |
| 10 | 863 | 1066 | 564 | 690 |  | 932 | 1152 | 822 | 999 |  | 622 | 766 | 617 | 770 |  | 378 | 486 | 390 | 521 |  | 225 | 238 | 237 | 250 |
| 11 | 828 | 1024 | 540 | 661 |  | 897 | 1110 | 787 | 958 |  | 585 | 724 | 584 | 731 |  | 348 | 452 | 350 | 475 |  | 180 | 191 | 190 | 201 |
| 12 | 828 | 1024 | 540 | 661 |  | 897 | 1109 | 787 | 958 |  | 585 | 724 | 583 | 730 |  | 347 | 451 | 350 | 474 |  | 179 | 191 | 190 | 201 |
| 13 | 689 | 861 | 451 | 557 |  | 743 | 929 | 653 | 799 |  | 423 | 542 | 447 | 576 |  | 227 | 318 | 156 | 248 |  | - | - | - | - |
| 14 | 49 | 66 | 16 | 24 |  | 65 | 94 | 57 | 73 |  | -7 | 8 | -9 | 9 |  | - | - | - | - |  | - | - | - | - |
| 15 | 56 | 95 | 66 | 99 |  | 32 | 63 | 0 | 20 |  | 15 | 92 | 210 | 305 |  | 127 | 301 | -104 | 104 |  | - | - | - | - |
| 16 | 366 | 460 | 256 | 319 |  | 381 | 482 | 311 | 388 |  | 303 | 419 | 456 | 590 |  | 352 | 541 | 233 | 484 |  | 323 | 340 | 340 | 356 |
| 17 | 81 | 98 | 58 | 69 |  | 81 | 99 | 80 | 98 |  | 80 | 105 | 75 | 94 |  | 67 | 84 | 90 | 112 |  | 106 | 110 | 110 | 114 |
| 18 | 81 | 98 | 58 | 70 |  | 80 | 100 | 80 | 98 |  | 75 | 111 | 73 | 96 |  | 65 | 87 | 89 | 114 |  | 106 | 110 | 111 | 115 |
| 19 | 0 | 0 | 0 | 0 |  | 68 | 76 | 90 | 103 |  | 1456 | 1660 | 1831 | 2178 |  | 2383 | 2815 | 2686 | 3255 |  | 9050 | 9138 | 9489 | 9600 |
| 20 | 0 | 0 | 0 | 0 |  | 0 | 0 | 0 | 0 |  | 0 | 1 | 0 | 1 |  | 0 | 1 | 0 | 1 |  | 1047 | 1053 | 1111 | 1121 |
| 21 | 679 | 846 | 539 | 668 |  | 817 | 1023 | 774 | 958 |  | 352 | 451 | 475 | 608 |  | 340 | 467 | 355 | 560 |  | 180 | 191 | 190 | 202 |
| 22 | 490 | 618 | 432 | 541 |  | 595 | 749 | 582 | 728 |  | 193 | 263 | 344 | 448 |  | 217 | 335 | 158 | 334 |  | - | - | - | - |
| 23 | 1016 | 1246 | 691 | 842 |  | 1069 | 1306 | 966 | 1170 |  | 841 | 1009 | 817 | 992 |  | 553 | 689 | 619 | 795 |  | 489 | 511 | 513 | 535 |
| 24 | 81 | 98 | 58 | 70 |  | 148 | 177 | 169 | 201 |  | 1525 | 1776 | 1903 | 2276 |  | 2446 | 2904 | 2774 | 3371 |  | 9160 | 9245 | 9604 | 9712 |
| 25 | 1264 | 1285 | 860 | 875 |  | 1346 | 1369 | 1268 | 1279 |  | 1955 | 1972 | 2090 | 2143 |  | 2146 | 2229 | 2605 | 2752 |  | 6173 | 6209 | 6490 | 6520 |
| 26 | 0 | 0 | 0 | 0 |  | 69 | 75 | 91 | 102 |  | 1461 | 1654 | 1834 | 2175 |  | 2386 | 2812 | 2687 | 3253 |  | 9050 | 9138 | 9489 | 9600 |
| 27 | 0 | 0 | 0 | 0 |  | 0 | 0 | 0 | 0 |  | 0 | 0 | 0 | 0 |  | 0 | 0 | 0 | 0 |  | 0 | 0 | 0 | 0 |
| 28 | 0 | 0 | 0 | 0 |  | 0 | 0 | 0 | 0 |  | 0 | 0 | 0 | 0 |  | 0 | 0 | 0 | 1 |  | 1046 | 1052 | 1111 | 1121 |
| 29 | 248 | 373 | 177 | 255 |  | 252 | 389 | 245 | 371 |  | 238 | 439 | 188 | 413 |  | 173 | 371 | 304 | 536 |  | 413 | 438 | 436 | 462 |
| 30 | 32 | 48 | 23 | 33 |  | 32 | 50 | 32 | 48 |  | 31 | 56 | 24 | 53 |  | 22 | 48 | 39 | 69 |  | 53 | 56 | 54 | 61 |
| 31 | 32 | 33 | 24 | 24 |  | 31 | 32 | 32 | 33 |  | 32 | 32 | 30 | 30 |  | 26 | 27 | 29 | 30 |  | 43 | 45 | 45 | 48 |
| 32 | 82 | 82 | 60 | 60 |  | 79 | 79 | 82 | 82 |  | 80 | 81 | 75 | 76 |  | 58 | 86 | 79 | 113 |  | 101 | 105 | 105 | 109 |
| 33 | 59 | 77 | 43 | 53 |  | 58 | 77 | 57 | 77 |  | 57 | 84 | 53 | 76 |  | 52 | 62 | 69 | 83 |  | 80 | 83 | 84 | 86 |
| 34 | 117 | 140 | 80 | 96 |  | 124 | 148 | 116 | 138 |  | 125 | 143 | 110 | 129 |  | 97 | 114 | 133 | 160 |  | 143 | 148 | 149 | 154 |
| 35 | 82 | 98 | 58 | 69 |  | 82 | 98 | 81 | 97 |  | 86 | 99 | 77 | 92 |  | 69 | 82 | 91 | 110 |  | 106 | 109 | 110 | 114 |
| 36 | 35 | 42 | 25 | 30 |  | 35 | 42 | 34 | 41 |  | 37 | 42 | 33 | 39 |  | 29 | 35 | 39 | 47 |  | 45 | 46 | 47 | 48 |
| 37 | 203 | 245 | 144 | 174 |  | 204 | 246 | 202 | 242 |  | 214 | 248 | 193 | 229 |  | 173 | 205 | 228 | 275 |  | 264 | 273 | 275 | 285 |
| 38 | 137 | 164 | 88 | 105 |  | 153 | 181 | 134 | 158 |  | 161 | 182 | 134 | 155 |  | 116 | 137 | 190 | 228 |  | 179 | 190 | 189 | 200 |
