# Supplementary material for: Oxygen dependence of metabolic fluxes and energy generation of Saccharomyces cerevisiae CEN.PK113-1A
Source: BMC Syst Biol. 2008 Jul 9;2:60. doi: 10.1186/1752-0509-2-60 (PMC2507709; doi:10.1186/1752-0509-2-60)
Supplement: Additional file 4 — Stoichiometric model of the central carbon metabolism of S. cerevisiae. Reactions in the stoichiometric model of the central carbon metabolism of Saccharomyces cerevisiae, including also anabolic fluxes from metabolic intermediates to biosynthesis, transport reactions across the mitochondrial membrane and uptake and excretion reactions, applied in the 13C-MFA determination of the metabolic net fluxes in different oxygenation conditions. [file 1752-0509-2-60-S4.doc]

x_i_ reaction

x_1_ ATP + glucose => G6P + ADP

x_2_ G6P => F6P

x_3_ G6P + 2*NADP^+^ => R5P +2* NADPH + CO_2_

x_4_ F6P + ATP => 2*G3P + ADP

x_5_ 2*R5P => S7P + G3P

x_6_ E4P + R5P => F6P + G3P

x_7_ S7P + G3P => F6P + E4P

x_8_ G3P + ADP + NAD^+^ => Pep + ATP + NADH

x_9_ Pep + ADP => ATP + Pyr_cyt_

x_10_ CoA + NAD+ + Pyr_mit_ => AcCoA_mit_ + NADH + CO_2_

x_11_ AcCoA_mit_ + Oaa_mit_ => CoA + citrate

x_12_ citrate + NAD^+^ => NADH + CO_2_ + Oga

x_13_ 2*NADH + Oga + GDP + FADH_2_ => Oaa_mit_ + 2*NAD^+^ + FAD + GTP + CO_2_

x_14_ Oaa_mit_ + NADP^+^ => NADPH + CO_2_ + Pyr_mit_

x_15_ ATP + Oaa_cyt_ => Pep + ADP + CO_2_

x_16_ ATP + CO_2_ + Pyr_cyt_ => Oaa_cyt_ + ADP

x_17_ CoA + ATP + acetate => AcCoA_cyt_ + Pyrophosphate + ADP

x_18_ AcO + NADP^+^ => NADPH + acetate

x_19_ AcO + NADH => Ethanol + NAD^+^

x_20_ G3P + NADH + ATP => glycerol + Orthophosphate + ADP + NAD^+^

x_21_ Oaa_cyt_ => Oaa_mit_

x_22_ Oaa_mit_ => Oaa_cyt_

x_23_ Pyr_cyt_ => Pyr_mit_

x_24_ Pyr_cyt_ => AcO + CO_2_

x_25_ glucose_ext_ => glucose

x_26_ ethanol => ethanol_ext_

x_27_ acetate => acetate_ext_

x_28_ glycerol => glycerol_ext_

x_29_ G6P => G6P_ext_

x_30_ R5P => R5P_ext_

x_31_ E4P => E4P_ext_

x_32_ G3P => G3P_ext_

x_33_ Pep => Pep_ext_

x_34_ Oaa_cyt_ => Oaa_ext_

x_35_ AcCoA_cyt_ => AcCoA_ext_

x_36_ AcCoA_mit_ => AcCoA_ext_

x_37_ Pyr_mit_ => Pyr_ext_

x_38_ Oga => Oga_ext_
